# Do marginal plant populations enhance the fitness of larger core units under ongoing climate change? Empirical insights from a rare carnation

**DOI:** 10.1093/aobpla/plac022

**Published:** 2022-05-12

**Authors:** Domenico Gargano, Liliana Bernardo, Simone Rovito, Nicodemo G Passalacqua, Thomas Abeli

**Affiliations:** Dipartimento di Biologia, Ecologia e Scienze della Terra dell’Università della Calabria, Via P. Bucci, I-87036 Arcavacata di Rende, Italy; Museo di Storia Naturale della Calabria ed Orto Botanico dell’Università della Calabria, loc. Polifunzionale, I-87036 Arcavacata di Rende, Italy; Dipartimento di Biologia, Ecologia e Scienze della Terra dell’Università della Calabria, Via P. Bucci, I-87036 Arcavacata di Rende, Italy; Museo di Storia Naturale della Calabria ed Orto Botanico dell’Università della Calabria, loc. Polifunzionale, I-87036 Arcavacata di Rende, Italy; Dipartimento di Biologia, Ecologia e Scienze della Terra dell’Università della Calabria, Via P. Bucci, I-87036 Arcavacata di Rende, Italy; Dipartimento di Biologia, Ecologia e Scienze della Terra dell’Università della Calabria, Via P. Bucci, I-87036 Arcavacata di Rende, Italy; Museo di Storia Naturale della Calabria ed Orto Botanico dell’Università della Calabria, loc. Polifunzionale, I-87036 Arcavacata di Rende, Italy; Department of Science, University of Roma Tre, Viale Guglielmo Marconi 446, 00146 Roma, Italy

**Keywords:** Assisted gene flow, climate change, *Dianthus guliae*, genetic rescue, inbreeding depression, maladaptation, marginal populations

## Abstract

Assisted gene flow (AGF) can restore fitness in small plant populations. Due to climate change, current fitness patterns could vary in the future ecological scenario, as highly performant lineages can undergo maladaptation under the new climatic contexts. Peripheral populations have been argued to represent a potential source of species adaptation against climate change, but experimental evidence is poor. This paper considers the consequences of within- and between-population mating between a large core population and the southernmost population, the rare *Dianthus guliae*, to evaluate optimal AGF design under current and future conditions. We performed experimental self-pollinations and within- and between-population cross-pollinations to generate seed material and test its adaptive value to aridity. Seed germination, seedling growth and survival were measured under current and expected aridity. Effects of population type, pollination treatment and stress treatment on fitness components were analysed by generalized linear models. Relative measures of inbreeding depression and heterosis were taken under different stress treatments. Self-pollination reduced fitness for all the considered traits compared to within- and between-population cross-pollination. Under current aridity regime, the core population expressed higher fitness, and a larger magnitude of inbreeding depression. This indicated the core unit is close to its fitness optimum and could allow for restoring the fitness of the small peripheral population. Contrarily, under increased aridity, the fitness of outbred core lineages decreased, suggesting the rise of maladaptation. In this scenario, AGF from the small peripheral population enhanced the fitness of the core unit, whereas AGF from the core population promoted a fitness loss in the peripheral population. Hence, the small peripheral population could improve fitness of large core units versus climate change, while the contrary could be not true. Integrating reciprocal breeding programmes and fitness analyses under current and predicted ecological conditions can support optimal AGF design in a long-term perspective.

## Teaser

The environmental variations due to ongoing climate change can reduce the reproductive success of species and populations that currently show a good conservation status. Through experiments of assisted gene flow between a small peripheral population and a large core population of a rare carnation, this paper evidences that small populations isolated at the margin of the species range can improve the reproductive success of larger demographic units under climate change. Such findings highlight the importance of preserving small peripheral populations as they can enhance the species evolutionary potential versus climate change.

## Introduction

Plant populations isolated at the margin of the geographic range can greatly contribute to local biodiversity and to the evolutionary potential of species (e.g. Pfeifer *et al.* 2010; Kyrkjeeide *et al.* 2020); therefore, they often inspired the interest of conservation and evolutionary biologists. The effects of geographical marginality vary substantially across species (Pfeifer *et al.* 2009; Abeli *et al.* 2015), as a function of the ecological relationships between central and peripheral sites (i.e. coincidence of geographical and ecological marginality), and of the investigated traits of population fitness (Dixon *et al.* 2013; Villellas *et al.* 2013). Nonetheless, large recognitions on empirical evidence suggest that geographical marginality tends to coincide with ecological and physiological marginality in the context of the species niche (Lee-Yaw *et al.* 2016; Abeli *et al.* 2020). This has relevant implications for the conservation and evolutionary role of peripheral populations under current trends of global environmental changes.

The viability of small peripheral populations often results from the effects of and the interplay between ecology (e.g. Gargano *et al.* 2007; Gerst *et al.* 2011), demography (e.g. Kanda *et al.* 2009; Gerst *et al.* 2011; Carbognani *et al.* 2019) and genetics (e.g. Kanda *et al.* 2009; Gargano *et al.* 2015; Birkeland *et al.* 2017). Indeed, the interplay between isolation and reduced population size can favour the rise of inbreeding depression (ID) and cause a severe loss of offspring viability (Leimu *et al.* 2006; Wright *et al.* 2008). The ongoing climate change (CC) is promoting significant global variations in temperature and precipitation regimes, and a substantial increase of the frequency of extreme climate events. Such climatic variations can exacerbate the fitness limitations commonly affecting the small and peripheral units, because inbred lineages often result more sensitive to environmental stress (Armbruster and Reed 2005; Cheptou and Donohue 2011; Fox and Reed 2011). Nonetheless, it has been argued that peripheral plant populations can represent a potential source of adaptation to climate-driven ecological variations because they are adapted to unique environmental conditions (Rehm *et al.* 2015). The hypothesis that patterns of plant adaptation to CC components may follow a geographical gradient across the species’ range is supported by an increasing body of experimental evidence. For instance, in *Silene suecica*, Abeli *et al.* (2015) found different responses in terms of plant growth under varying water availability between northern core populations and southern peripheral populations. Similar findings were detected in the timing of seedling emergence in the range-restricted and short-lived *Mimulus laciniatus*, which showed rapid adaptation to enhanced drought in seeds from low-elevation marginal populations (Dickman *et al.* 2019). Therefore, comparing the fitness patterns of core and peripheral populations under different climatic contexts can be helpful for understanding plant adaptation processes to a warmer climate (Abeli *et al.* 2018), and for designing effective conservation actions aiming to reduce current and future CC impact on biodiversity.

The conservation status of severely depleted populations can be enhanced by facilitating immigration from foreign large units (i.e. genetic rescue) (Frankham 2015). There is evidence that facilitated immigration increases genetic diversity, with benefits for the fitness of plant populations close to extinction (Willi and Fischer 2005; Finger *et al.* 2011; Oakley and Winn 2012). Benefits of genetic rescue in small peripheral populations can be achieved by introducing genes from large core demographic units through the translocation of individuals (i.e. assisted migration) or by favouring interpopulation mating via pollen transfer (i.e. assisted gene flow [AGF]). Assisted gene flow is an effective way for the genetic and fitness improvement of small and isolated plant populations (Aitken and Whitlock 2013; Gargano *et al.* 2015; Whiteley *et al.* 2015). However, benefits of genetic rescue may be limited by outbreeding depression (OD) and gene swamping (Tallmon *et al.* 2004). For instance, large divergence among populations makes more likely the rise of OD (Frankham *et al.* 2011; Pekkala *et al*. 2012; Whiteley *et al.* 2015). The patterns and magnitude of OD can significantly vary depending on the ecological context (Frankham *et al.* 2011; Whiteley *et al.* 2015). Maladaptation (i.e. the component of OD determined by the interaction between genome and environment) is predicted to have profound effect on species evolutionary (and conservation) trajectories under changing environments (Brady *et al.* 2019), since the novel ecological conditions determined by ongoing CC can substantially modify the current fitness patterns among populations. Accordingly, lineages adapted to current conditions may lose their fitness advantage under the effect of CC (Henry *et al.* 2003; Cheptou and Donohue 2011). The projected changes in the climate system depend on future greenhouse gasses emission scenarios. The increase of global mean surface temperature by the end of 21st century (2081–2100) relative to 1986–2005 could reach 4.8 °C under the most pessimistic scenario (IPCC 2014). In addition, in many mid-latitude regions the mean precipitation will likely decrease over the same timeframe (IPCC 2014). Therefore, the ongoing trends of ecological variation must be considered for planning AGF actions aiming at preserving small border populations. Indeed, the future fitness consequences of introducing genes from large core populations in small peripheral units may be different than expected under current conditions. In contrast, when the interplay of geographical and ecological marginality has promoted the selection of adaptive traits against CC, small border populations may become the source for restoring fitness of large and viable demographic units under the novel climatic scenario.

In this study, we investigated the fitness patterns induced by self-pollination, within-population cross-pollination and between-population cross-pollination under current and increased environmental aridity in a core and in a peripheral population of the Italian endemic *Dianthus guliae*. Previous work carried out under neutral ecological conditions revealed that the peripheral population suffers from strong reduction of genetic diversity and reduction of fitness and could benefit from genetic rescue through AGF (Gargano *et al.* 2015). In this paper, we evaluate if such a genetic rescue benefit is maintained under experimental conditions simulating the trend of increasing aridity affecting the southern edge of the species range (Federico *et al*. 2009, 2010). To this end, we analysed two early fitness components (i.e. seed germination and post-germination survival) and two phenotypic traits that can affect fitness (i.e. seedling radicle length and shoot size) in lineages representing different combinations of within- and between-population crossing under current versus arid conditions. We aimed to answering the following questions: (i) how do different drivers (i.e. demographic condition, mating pattern and ecological context for germination and early growth) affect early fitness components, namely germination, post-germination survival, seedling radicle length and shoot size? (ii) does an increase in aridity stress exacerbate fitness differences between inbred and outbred lineages? (iii) does the peripheral population of *D. guliae* display higher adaptation to aridity? and (iv) does AGF remain an effective option for preserving the adaptive potential of the species as a whole under ongoing CC?

## Materials and Methods

### Study species

**Figure 1.**
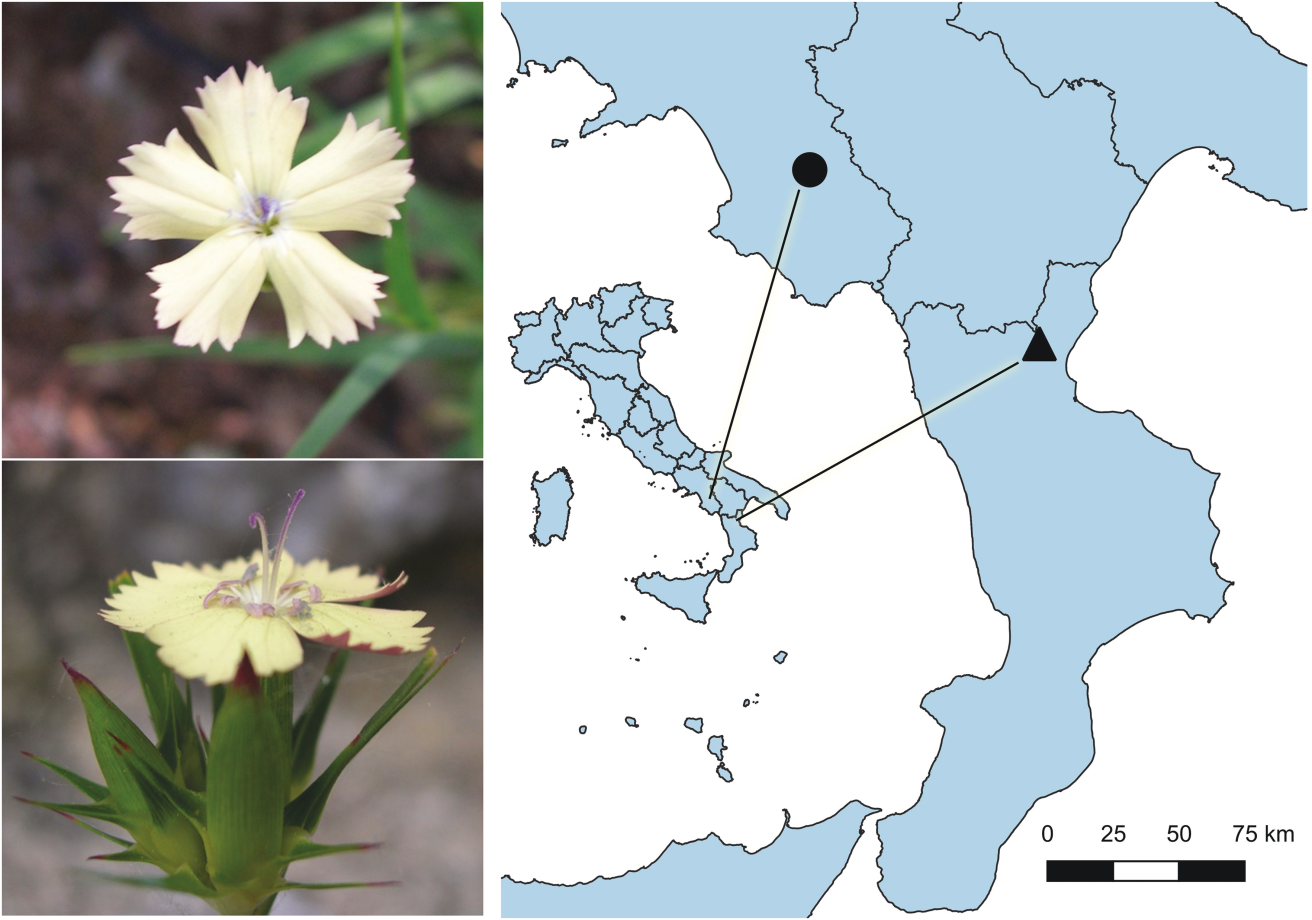
Upper and lateral views of hermaphrodite flowers of *Dianthus guliae* (left-hand photos) and location of the studied populations in the context of the Italian peninsula (right-hand map). Core population, black dot; peripheral population, black triangle.

*Dianthus guliae* is a perennial herb with numerous stems bearing terminal heads of 2–8 flowers, the upper side of the corolla varies from citrine to orange-yellow (Peruzzi and Gargano 2006) (Fig. 1). Due to male sterility, the populations of this protandrous plant host individuals belonging to three sexual morphs: hermaphrodite (i.e. plants bearing only perfect flowers), female (i.e. plants bearing only pistillate flowers) and mixed (i.e. plants bearing both perfect and pistillate flowers) (Gargano *et al.* 2009). The species’ pollination system involves a suite of butterflies (e.g. *Thymelicus sylvestris*, *Macroglossum stellatarum*, *Papilio machaon*) and *Hadena* nocturnal moths (Gargano *et al.* 2009). As found for the related *D. balbisii* (Gargano *et al.* 2017), such a generalist pollination syndrome would enhance reproduction in the heterogeneous habitat of the plant (Gargano 2011). Seeds of *D. guliae* lack adaptations to specialized dispersal syndromes; their dispersion appears mainly due to barochory or boleochory. *Dianthus guliae* is fully self-compatible and, despite male sterility and dichogamy, self-fertilization can occur through geitonogamy (Gargano *et al.* 2009). However, after self-fertilization, the species often undergoes severe and long-lasting ID (Gargano *et al*. 2009, 2011). *Dianthus guliae* inhabits borders and clearings of *Quercus cerris* forests at elevation close to 900–1000 m a.s.l. The species’ habitat is threatened by deforestation and land-use changes (Gargano 2011). Consequently, over the 20th century, the plant disappeared from several stands and, currently, it is found in a few stands in the Cilento National Park and in the Pollino National Park (Fig. 1). The greatest number of populations (~10) is confined into a small area of the Cilento National Park, with a generally small (*N* < 100) to very small (*N* < 50) population size. At the southern range periphery, there is a unique isolated population (<50 individuals) severely threatened by genetic drift and ID (Gargano *et al.* 2015). Due to the continuous decline in range size and habitat quality, *D. guliae* qualified as endangered in the red list of the vascular flora endemic to Italy (Orsenigo *et al.* 2018).

### Seed source and genetics

**Figure 2.**
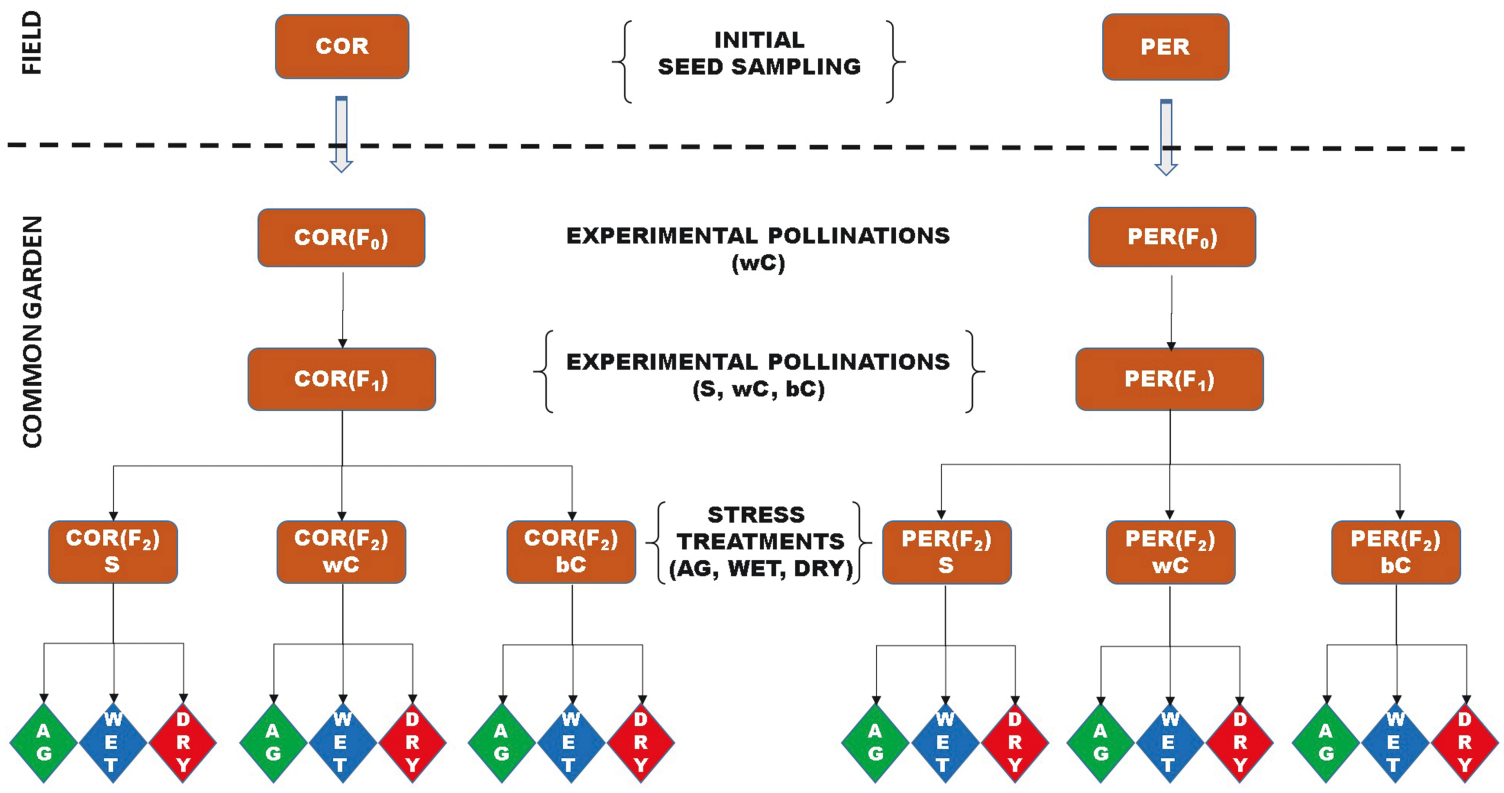
Schematic representation of the experimental workflow, from the initial seed sampling in the field to the subsequent stages carried out in common garden, aiming to obtain an array of plants representing different combinations of population types and pollination treatments for performing fitness analyses under varying stress levels. Legend for pollination and stress treatments: S = self-pollination; wC = within-population cross-pollination; bC = between-population cross-pollination; AG = germination on agar; WET = germination and growth under current climatic conditions; DRY = germination and growth under increased aridity.

A schematic illustration of the experimental workflow is provided in Fig. 2. Initially, a collection of seeds was sampled in a relatively large core population (COR) and in the small population isolated at the southern margin of the species range (PER) (Fig. 1). In both the collection sites, *D. guliae* inhabits the edge of *Q. cerris* forests. In spite of the elevation gap, the data available at worldclim.org (Hijmans *et al.* 2005) indicate the two sites are quite similar from a climatic viewpoint, with a more pronounced summer aridity in PER. Indeed, COR occurs at 600 m a.s.l., annual mean temperature is 12.4 °C, annual precipitation is 866 mm and the precipitation of the driest month accounts for 25 mm. Instead, PER occurs at 870 m a.s.l., annual mean temperature is 12.2 °C, annual precipitation accounts for 885 mm and the precipitation of the driest month is 19 mm. Previous work (Gargano *et al.* 2015) revealed a substantial difference of heterozygosity between the two populations (*H*_COR_ = 0.68, *H*_PER_ = 0.38).

At the end of summer 2010, in each population, 40–50 seeds were collected on more than 20 plants growing at least 10 m apart. According to the germination and cultivation protocols described in Gargano *et al.* (2015) (**see****Supporting Information—Appendix S1** for details), the seeds collected in the field were used to obtain a set of F_0_ plants. The F_0_ plants were subjected to a preliminary run of experimental crosses aiming to create an array of experimental plants with reduced effects of the maternal growth context. Therefore, in 2011, 20 F_0_ plants (representing 20 different families from the source population) per each population were isolated before blooming, and then subjected to experimental cross-pollinations with a different plant of the same population (Fig. 2). On F_0_ plants, a variable amount of flowers was manually pollinated by brushing one dehiscent anther from two randomly selected plants against each stigma branch. The flowers used to collect pollen were not considered as receivers. When fruits were ripened, F_1_ seeds were collected and treated as described above to obtain adult plants for further experiments. In the 2012 flowering season, 15 F_1_ plants per population were selected and subjected to three pollination treatments: self-pollination (S), within-population cross-pollination (wC) and between-population cross-pollination (bC). The wC and bC treatments were performed by adopting the same procedure used for cross-pollinations on F_0_ plants. As reported in Gargano *et al.* (2015), this allowed producing six F_2_ lineages (Fig. 2) characterized by marked differences of genetic richness (Table 1). Once fruits ripened, a sample of seeds was collected on plants that produced offspring under each pollination treatment. More specifically, 5 plants (families hereafter) per population produced matched offspring, allowing to collect 600 seeds (~20 seeds × pollination treatment × family) for subsequent fitness analyses.

**Table 1.** Heterozigosity of the six F_2_ lineages produced by the experimental pollinations performed on plants from the core and peripheral population (data from Gargano *et al.* 2015). S = self-pollination; wC = within-population cross-pollination; bC = between-population cross-pollination.

Population type	Pollination treatment	Heterozigosity (%)
CORE	S	0.62
	wC	0.72
	bC	0.65
PERIPHERAL	S	0.28
	wC	0.40
	bC	0.54

### Experimental germination and seedling cultivation

The sampled F_2_ seeds were stored at 4 °C for 2 months. Afterwards, the seeds of each lineage were split among three treatments simulating a varying stress level (Fig. 2). Such stress treatments were designed according to the decreasing precipitation trend recently detected over the species’ range (Federico *et al*. 2009, 2010). The effect of AGF under the simulated CC scenario was evaluated by analysing the rates of germination and post-germination survival/growth in offspring representing different combinations of population type (i.e. COR and PER) and pollination treatment (i.e. S, wC and bC) under each stress treatment. In the first stress treatment (treatment code = AG), representing the optimal conditions for germination, 160 seeds were germinated in Petri dishes on agar 1 % at 18 °C. In the other treatments, it was used a larger seed sample (*N* = 240) in order to increase the availability of seedlings for measuring post-germination fitness proxies. The second treatment (treatment code = WET) was designed to represent the current field conditions during the germination season. Then, each seed was sown in a 3.5 × 3.5 cm pot filled with brown soil, daily irrigated with distillate water up to reach an amount of 100 mL × month. According to current precipitation regime, this treatment matched with 81.6 mm of monthly precipitation, calculated as *P* = (*W*/*a*^2^) * 10; where *P* = precipitation in mm, *W* = monthly supplied water in mL, *a* = irrigated surface in cm^2^. Such a reference precipitation value was obtained from current (1960–90) WorldClim data set (Hijmans *et al.* 2005). The third stress treatment (treatment code = DRY) simulated a scenario of increasing aridity. It consisted in reducing by 20 % the monthly supplied water, as deduced by the precipitation trends recorded in S-Italy over the last decades (Federico *et al*. 2009, 2010). Hence, the DRY treatment was designed to mirror actual trends of increasing aridity over the range of the study species. In this treatment, each seed was sown in a 3.5 × 3.5 cm pot filled with brown soil, daily irrigated with distillate water up to reach an amount of 80 mL × month, corresponding to 65.3 mm of monthly precipitation.

Because previous work on this species revealed high germination rates in seeds collected just after fruit ripening (Gargano *et al*. 2011, 2015), in these experiments it was assumed that most of *D. guliae* seeds germinate between the end of the summer and beginning of the autumn. Accordingly, for both WET and DRY treatments, the amount of monthly supplied water was determined based on the average monthly precipitation expected in the PER site during the 4 months (August–November) following dispersal.

### Estimating rates of germination, early growth and survival

The fitness proxies considered in this study included germination and post-germination traits. Then, after sowing, Petri dishes and pots were checked daily to record germination in each seed lineage under the three stress treatments. In the AG treatment, seeds were considered as germinated once we detected the appearance of radicle, while in WET and DRY treatments, seeds were considered as germinated once we detected the first signals of the emergence of cotyledons. Indeed, in the latter two protocols, the seeds were placed 1–2 mm below the soil surface; hence, the early germination phase (radicle emergence) was not visible. Since radicle and subsequent cotyledon emergence are closely related and are separated by a short interval, the two approaches adopted to check germination were expected to produce highly comparable results. To measure post-germination traits (only WET and DRY treatments) the seedlings were followed for 21 days. Over this period, seedling mortality was annotated daily. At the end of the observation period, the survived plants were uprooted. Subsequently, each seedling was carefully washed with distillate water in order to measure the radicle length and shoot size.

### Data analyses

The described procedures allowed gaining data about four early fitness components under different combinations of population type, pollination and stress treatment. Such fitness proxies included two binary fitness traits, probability of seed germination (GERM) and probability of post-germination survival (SURV), and two continuous phenotypic traits related to fitness, radicle length (ROOT, mm) and shoot size (SHOOT, mm). The effect of population type, pollination treatment and stress treatment on the four fitness proxies was evaluated by means of generalized linear models (GzLMs). Such models included the population type, the pollination treatment and the stress treatment as main effects. Moreover, the possible influence of mother plants on offspring fitness was evaluated by incorporating a further term accounting for the maternal family as a variable nested within the population type. The models carried out on binary response variables (GERM and SURV) were ran with three different link functions: logit, probit and complementary log–log. Instead, in the models including continuous response variables (ROOT and SHOOT), two alternative link functions were applied: identity (data not transformed) and log (log-transformed data). In all cases, the model with best performance was determined according to the finite sample corrected Akaike’s information criterion (AICC) **[see****Supporting Information—Table S2.1****]**. The likelihood-ratio chi-square between the adapted model versus the null (i.e. intercept) model was applied as a global test of significance for all the models described above. Pairwise comparisons for each term were carried out based on the Bonferroni *post hoc* test.

The GzLMs on GERM were carried by considering 600 seeds representing different families (58–61 seeds per 10 families), population types (COR = 300; PER = 300), pollination treatments (S = 200; wC = 200; bC = 200) and stress treatments (AG = 160; WET = 220; DRY = 220). The analysis on SURV involved 480 seedlings representing different families (47–49 seedlings per 10 families), population types (COR = 240; PER = 240), pollination treatments (S = 160; wC = 161; bC = 159) and stress treatments (WET = 240; DRY = 240). Finally, the effect of family, population type, pollination treatment and stress treatment on ROOT and SHOOT was evaluated on 306 seedlings representing different families (26–33 seedlings per 10 families), population types (COR = 162; PER = 144), pollination treatments (S = 83; wC = 111; bC = 112) and stress treatments (WET = 162; DRY = 144).

In order to evidence the possible environmental impact on the fitness induced by self-pollination and within- and between-population cross-pollination, all the fitness components were used to perform relative measures of ID and heterosis (*H*) under each stress treatment. Therefore, ID was calculated as:


$$\begin{array}{l} ID=1-(W_{selfed}/W_{outbred}) \end{array}$$

being *W*_selfed_ and *W*_outbred_ the mean fitness value expressed for a given trait, respectively, by the selfed and outcrossed progeny within a population. Values of ID > 0 evidenced a fitness loss in the inbred offspring compared to the outbred one, suggesting the rise of ID. Instead, values of ID < 0 indicated a reduced fitness in the outbred offspring compared to the inbred one, as a possible consequence of OD.

Likewise, the measures of heterosis were made as:


$$\begin{array}{l} ID=1-(W_{selfed}/W_{outbred}) \end{array}$$

being *W*_outbred_ and *W*_hybrid_ the mean fitness value expressed for a given trait, respectively, by the outcrossed progeny within a population and the ‘hybrid’ progeny produced by between-population mating. Values of *H* > 0 indicated that between-population cross-pollination provided a fitness benefit compared to within-population cross-pollination, by supporting the effectiveness of AGF. Contrarily, values of *H* < 0 evidenced a fitness loss after between-population cross-pollination compared to within-population cross-pollination, by suggesting the rise of OD and, then, that AGF could be counterproductive. The calculated values of ID and *H* between population types and among stress treatments are provided in **Supporting Information—Table S2.2**.

The numerical data sets relative to the above-described analyses are provided in **Supporting Information—Appendix S3**.

## Results

### Patterns of seed germination

**Figure 3.**
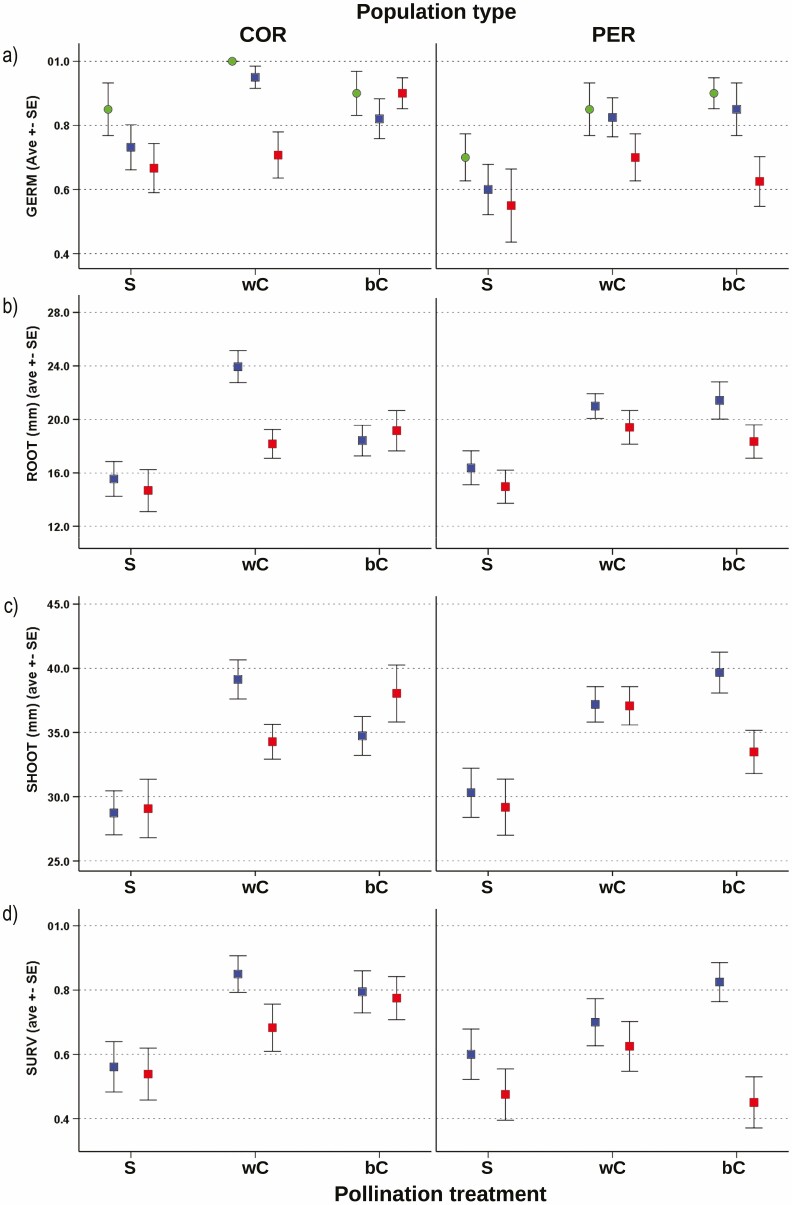
Average percent of seed germination (A), seedling radicle length (B), seedling shoot size (C) and percent of seedling survival (D) across different pollination and stress treatments in the core and peripheral population of *D. guliae*. Legend for pollination treatments: S = self-pollination; wC = within-population cross-pollination; bC = between-population cross-pollination. Symbols for stress treatments: Green circles = AG; left-hand blue squares = WET; right-hand red squares = DRY.

Based on AICC scores, the complementary log–log transformation of GERM data produced the model with the best performance **[see****Supporting Information—Table S2.1****]**. The analysis evidenced a significant overall variation of seed germination (likelihood-ratio chi-square = 52.653; df = 21; *P* < 0.001). The maternal family exhibited a negligible influence (Table 2), suggesting absence of relevant patterns across offspring of different mother plants in each population **[see****Supporting Information—Fig. S4.1****]**. Instead, the percentage of seed germination significantly varied between population types (Table 2). The average germination rate in PER population was significantly lower than observed in COR (respectively, 74.0 % and 86.0 %; *P* = 0.001) (Fig. 3A). Significant differences in seed germination also occurred among pollination treatments (Table 2), and denoted a similar pattern in both the demographic units (Fig. 3A). The self-pollination induced lower seed germination than within-population cross-pollination (68.0 vs. 86.0 %; *P* = 0.001), and between-population cross-pollination (68.0 vs. 86.0 %; *P* = 0.001). Instead, within- and between-population cross-pollination did not show significant differences (*P* = 1.000). A significant effect was also due to the stress treatment (Table 2). As expected, the germination rate under the DRY treatment (69.0 %) was lower than found in AG (89.0 %; *P* < 0.001) and WET (80.0 %; *P* = 0.04), while no significant differences occurred between the latter two treatments (*P* = 0.09). Finally, no significant interactions were detected between main effects (Table 2).

**Table 2.** Effects of different combinations of population type, and pollination and stress treatment on germination and post-germination fitness components.

Source of variation	GERM			ROOT			SIZE			SURV		
	df	Wald χ^2^	*P*	df	Wald χ^2^	*P*	df	Wald χ^2^	*P*	df	Wald χ^2^	*P*
Population type	1	11.039	0.001	1	0.011	0.917	1	0.032	0.857	1	4.447	0.035
Family (Population type)	8	6.542	0.587	8	3.657	0.887	8	5.073	0.750	8	2.860	0.943
Pollination treatment	2	19.607	<0.001	2	25.796	<0.001	2	36.640	<0.001	2	14.723	<0.001
Stress treatment	2	18.345	<0.001	1	6.749	0.009	1	2.054	0.152	1	9.540	0.002
Population type * Pollination treatment	2	0.018	0.991	2	2.459	0.293	2	0.215	0.898	2	2.089	0.352
Population type * Stress treatment	2	0.235	0.889	1	0.007	0.932	1	1.461	0.227	1	1.180	0.277
Pollination treatment * Stress treatment	4	3.809	0.432	2	3.154	0.207	2	1.114	0.573	2	1.981	0.371

**Figure 4.**
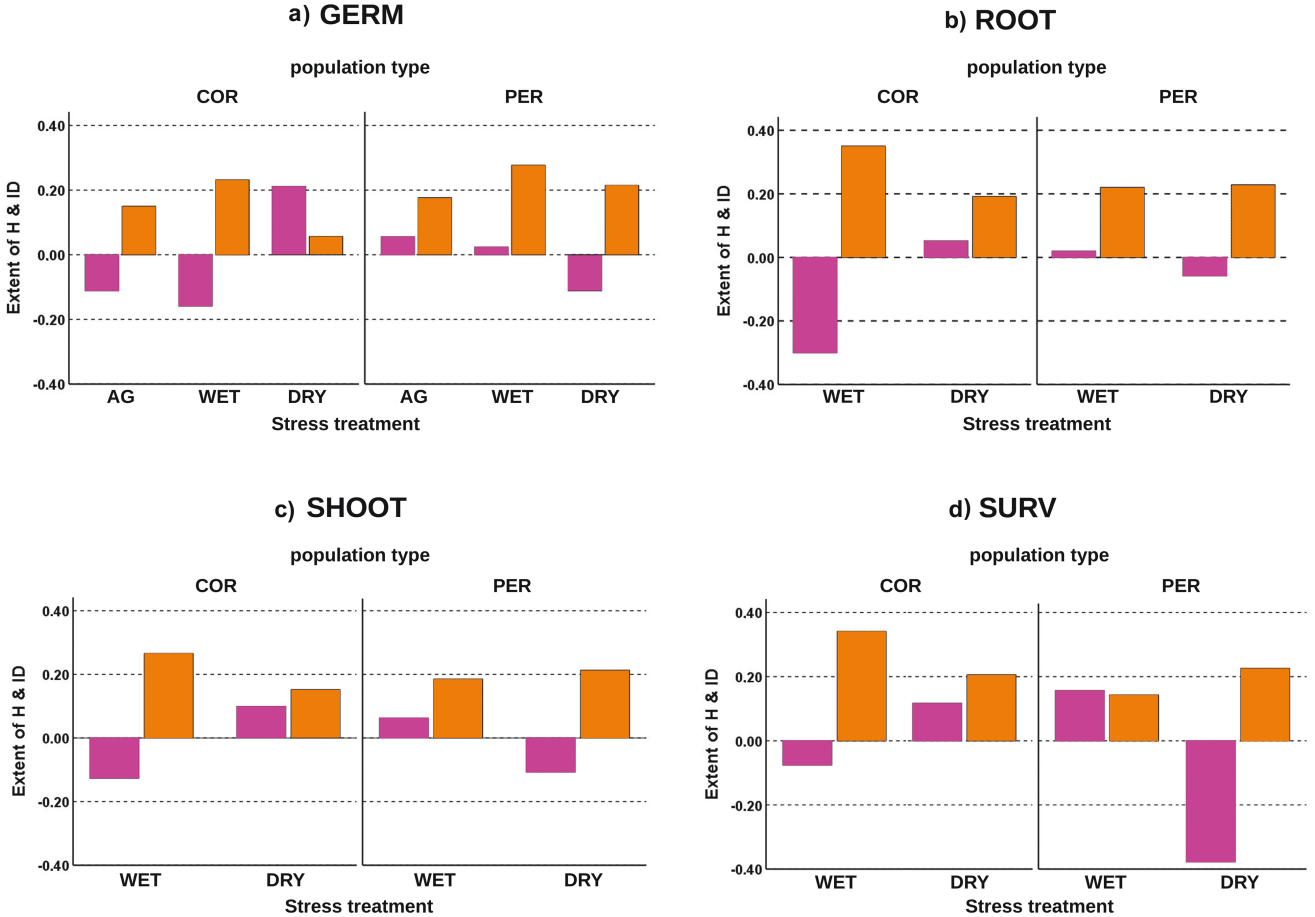
Patterns of heterosis (*H*, left-hand violet bars) and inbreeding depression (ID, right-hand yellow bars) on seed germination (A), seedling radicle (B), shoot (C) and survival (D) in the two studied populations under the three stress treatments. Positive values of ID indicate fitness loss after self-pollination for the rise of ID; negative values of ID evidence a fitness loss after within-population cross-pollination that could be a consequence of OD. Positive values of H indicate heterosis benefit provided by between-population AGF; instead, negative values of H suggest the rise of maladaptation after between-population AGF. Numerical values of H and ID are supplied in **Supporting Information—Table S2.2**.

Compared to self-pollination, within-population cross-pollination favoured germination under all stress treatments in both populations (Fig. 3A), resulting in positive values of ID (Fig. 4A). The relative measure of seed germination suggested that the stress treatment had some effect on the levels of ID detected in the two populations. While the two populations showed a similar increase of ID from the AG to the WET stress treatment (Fig. 4A), the DRY stress treatment induced a reduction of ID in COR and no evident variation in PER (Fig. 4A). This was due to a reduction of seed germination occurred under the DRY treatment in the COR offspring produced by within-population cross-pollination (Fig. 3A). Likewise, the measures of heterosis evidenced that the effect of between-population cross-pollination on seed germination depended upon the population type and stress treatment. In the COR population, between-population cross-pollination caused a decline of seed germination under AG and WET treatments (Fig. 3A), resulting in negative heterosis (Fig. 4A). Instead, under the DRY treatment, interpopulation mating favoured seed germination, resulting in positive heterosis (Fig. 4A). In PER population, between-population cross-pollination increased seed germination in AG and WET, while it reduced germination in the DRY treatment (Fig. 3A). Therefore, in the peripheral unit, a weak positive heterosis occurred under AG and WET treatments, but such a heterosis benefit was lost under the DRY one (Fig. 4A).

### Patterns of early growth

According to AICC values, the model based on log-transformed values showed the best performance for both ROOT and SHOOT **[see****Supporting Information—Table S2.1****]**. The GzLM evidenced a significant overall variation of radicle length (likelihood-ratio chi-square = 48.627; df = 17; *P* < 0.001). Again, no evident variation occurred across the progeny of different mother plants within each population (Table 2; **see****Supporting Information—Fig. S4.2**). Most of variation originated from the pollination and stress treatment (Table 2); while the effects linked to the population type, and to any interaction was not significant (Table 2). Self-pollination caused a significant reduction of radicle length compared to within-population cross-pollination (15.4 vs. 20.3 mm; *P* < 0.001), and to between-population cross-pollination (15.4 vs. 19.3 mm; *P* < 0.001) in both the stress treatments (Fig. 3B). Instead, differences between within- and between-population cross-pollination were not significant (*P* = 0.917).

As far as the analyses on SHOOT are concerned (Fig. 3C; **see****Supporting Information—Fig. S4.3**), the model indicated a large variation of shoot size (likelihood-ratio chi-square = 49.024; df = 17; *P* < 0.001). Such variation was mainly explained by the pollination treatment, while other factors and interactions exerted no effects (Table 2). The average shoot size after self-pollination (29.3 mm) was significantly smaller than found in the other pollination treatments (within-population cross-pollination = 34.8 mm, *P* < 0.001; between-population cross-fertilization = 36.6 mm, *P* < 0.001) in both the stress treatments (Fig. 3C). As found for ROOT, no significant differences occurred between within- and between-population cross-pollination (*P* = 1.000).

The relative measures of ID and *H* evidenced similar patterns for both ROOT and SHOOT across populations and stress treatments (Fig. 4B and C). In COR, a reduction of ID occurred from the WET to the DRY treatment. It was mainly due to the decline of radicle and shoot size in offspring produced by within-population cross-pollination (Fig. 3B and C). In contrast, PER offspring evidenced little variation of ID between stress treatments. Furthermore, the populations evidenced reverse patterns of heterosis. COR showed negative or positive *H*, respectively, under the WET and DRY treatment, while PER showed an opposite pattern (Fig. 4B and C). Then, the relative fitness measures indicated that the demographic and stress context influenced the levels of ID and *H* on seedling radicle and shoot growth.

### Patterns of early survival

Based on AICC scores, the model adopting logit-transformed values of SURV possessed the best performance **[see****Supporting Information—Table S2.1****]**. Overall, the population type, pollination and stress treatment promoted a significant variation of early survival rates (likelihood-ratio chi-square = 35.508; df = 17; *P* = 0.005). Most of the observed variability depended on the pollination and stress treatment, while the population type had a lower, yet significant, influence (Table 2). Instead, the influence of maternal family and of any between-factor interaction was negligible (Table 2; **see****Supporting Information—Fig. S4.4**). The survival of PER offspring was lower than observed in COR (62.0 vs. 72.0 %; *P* = 0.03). As expected, the DRY treatment induced a striking reduction of survival rates (*P* = 0.002), especially in the selfed offspring of the peripheral population (Fig. 3D). The survival rate after self-pollination was significantly lower than found after within-population cross-pollination (54.0 vs. 72.0 %; *P* = 0.003) and between-population cross-pollination (54.0 vs. 73.0 %; *P* = 0.002) in both populations and stress treatments. On the contrary, within- and between-population cross-pollination promoted quite comparable survival rates (*P* = 1.000).

The relative measures of ID and heterosis evidenced some patterns between populations and stress treatments (Fig. 4D). According to the above-mentioned effect of self-pollination, the magnitude of ID was positive in both populations and stress treatments. However, while COR showed a decreasing ID from WET to DRY treatment, in PER the magnitude of ID varied in a reverse fashion (Fig. 4D). Such pattern of ID reflected the larger fitness reduction caused by the DRY treatment on the COR offspring representing within-population cross-pollination (Fig. 3D). Analogously, the populations evidenced reverse patterns of heterosis. The COR population showed negative or positive *H*, respectively, under WET and DRY treatment, while the PER population revealed an opposite pattern (Fig. 4D). Accordingly, under the DRY treatment, between-population cross-pollination determined a survival benefit in COR, while in the peripheral population the seedlings obtained by interpopulation mating underwent a loss of viability (Fig. 3D). Therefore, as found for the other fitness proxies, the relative fitness measures indicated that the demographic and stress conditions conditioned the patterns of ID and *H* on early survival.

## Discussion

### Comparing fitness drivers in the study system (Q1)

Our data allow evaluation of the relative contribution to some early fitness components (i.e. seed viability, early growth vigour and early survival) of three major drivers: demographic condition (i.e. large core vs. small peripheral unit), mating pattern (i.e. self-pollination, within-population cross-pollination and between-population cross-pollination) and ecological context (i.e. different drought stress for germination and subsequent growth). Overall, the response to the tested fitness drivers fitted expectations. The peripheral population showed lower fitness values, as expected in small and isolated populations that undergo a severe decline in fitness associated with low genetic diversity (e.g. Wright *et al.* 2008; Oakley and Winn 2012; Birkeland *et al.* 2017; Carbognani *et al.* 2019). Self-pollination significantly reduced levels of seed and seedling viability. This supports the hypothesis that outcrossing plants, like *D. guliae* (Gargano *et al*. 2009, 2011), are more prone to pervasive ID (e.g. Takebayashi and Delph 2000; Godefroid *et al.* 2014). Our data also highlighted that increasing aridity can affect the rate of seed germination and seedling vigour and survival. This is congruent with current knowledge about the limitations caused by drought-related osmotic stress on early plant life stages (Orsenigo *et al.* 2017; Rossi *et al.* 2020).

Our results pointed out that the pollination treatment induced the largest effect on all the measured fitness proxies. Instead, the stress treatment influenced three out of the four measured traits. Finally, the population type exerted higher influence on seed germination and minor effect on seedling growth and survival (Table 2). These findings are crucial, because they highlight the importance of preserving optimal pollination and growth contexts for both the population types. In spite of the substantial differences in terms of genetic richness (Gargano *et al.* 2015), the core and peripheral population of *D. guliae* displayed similar responses to the constraints induced by self-fertilization and increasing aridity. This suggests that the species has an overall low ability to cope with a reduction of pollinator and resource availability. The prominent effect of pollination treatment in determining the fitness patterns observed in our study system is consistent with the general expectation that the breeding system is a major modulator of plant fitness. Compared to mixed breeders, outcrossing species often undergo higher fitness loss with the increasing levels of inbreeding that can follow habitat reduction and/or fragmentation (Godefroid *et al.* 2014; Aguilar *et al.* 2019). The relevant role of cross-pollination for maintaining population fitness emphasizes the importance of preserving an optimal pollination context. This could be achieved by preventing habitat transformations that affect plant–pollinator interactions and cause quantitative and qualitative loss of reproductive efficiency (Gargano *et al.* 2017).

### Interplay between inbreeding, ID and increasing aridity stress (Q2)

Variation in environmental stress can influence the responses to inbreeding and outbreeding observed on specific traits (e.g. Mustajärvi *et al.* 2005). In our study system, the responses of each genetic lineage to stress treatments seemed to be quite similar across the measured traits (Fig. 3). The selfed lineages showed an overall fitness loss compared to ones obtained by within-population cross-pollination under each stress condition. This would suggest the rise of ID in all the contexts and for all the traits (Fig. 4). Such findings agree with the expectation that outcrossing plants tend to suffer severe ID in early traits (e.g. Mustajärvi *et al.* 2005; Collin *et al.* 2009). The increase in aridity induced a general loss of seed viability and seedling vigour and survival. Such a fitness loss under harsher germination and growth conditions was more pronounced in the inbred lineages compared to the outbred ones of the same population. The experimentally induced drought stress caused higher fitness costs in the inbred lineage of the peripheral population. Especially, our results showed that this lineage experienced a more severe loss of seed viability and seedling survival compared to the outbred offspring of the same population, and to inbred and outbred lines of the core demographic unit (Fig. 3). The enhanced fitness loss affecting the inbred offspring of the peripheral population under the dry scenario agrees with a previous work, in which inbred offspring of *D. guliae* revealed more severe fitness restrictions with increasing environmental stress (Gargano *et al.* 2011). Such findings fit the expectation that inbred offspring can be more constrained than outbred ones under harsher environmental conditions (e.g. Eckert and Barrett 1994; Ramsey and Vaughton 1998; Cheptou *et al.* 2000). As also supported by the higher ID showed by the peripheral population under more stressful treatments (Fig. 4), such findings could be at least partially explained by the occurrence of context-dependent ID. Indeed, a positive relationship between magnitude of ID and environmental severity typically occurs when the inbred lineages are more sensitive to environmental stress (Armbruster and Reed 2005; Cheptou and Donohue 2011; Fox and Reed 2011).

The reduction of ID on seed viability found in both populations under the DRY treatment (Fig. 4A) and, especially, the generally lower ID recorded in the core population under more severe treatment (Fig. 4) produced a more complex picture of the interplay between ID and environmental constraints in our study system. The relationship between ID and environmental severity is not straightforward, as it depends on how the loci associated with ID interact with the changing ecological parameters (Cheptou and Donohue 2011). In our case, the overall reduction of ID found in the core population under increasing drought stress was mainly determined by a fitness reduction of the outbred offspring. This would suggest that the fitness advantage possessed by the outbred offspring of *D. guliae* in benign contexts was lost in the stressful context. Such a pattern fits the expectation that lower ID under stress can rise when the outbred lineages have an enhanced ability for taking full fitness advantage in suitable environments (Henry *et al.* 2003; Cheptou and Donohue 2011). As a major implication of such findings, the current adaptive response observed in early life-cycle stages of inbred and outbred offspring of *D. guliae* could vary under the future ecological scenario shaped by CC. Especially, the populations that currently possess higher genetic diversity and fitness may undergo severe constraints.

### Occurrence of higher adaptation to aridity at the southern periphery of the species’ range (Q3)

The observed patterns of context-dependent ID suggest that the outcrossed progeny of the core population could meet a more pronounced relative fitness loss in more arid contexts. This suggests that the simulated environmental variations, along with the magnitude of ID, can also have influenced patterns of maladaptation in our study system. Indeed, when outbred lineages are well-adapted to benign conditions, they can result maladapted when novel and limiting environmental conditions appear, resulting in decreasing relative ID (Ronce *et al.* 2009; Cheptou and Donohue 2011). Such findings support the context dependency of OD (Frankham *et al.* 2011; Whiteley *et al.* 2015). Mainly, maladaptation (i.e. the OD determined by the interaction between genome and environment) is expected to exert increasing limitations in wild populations because of the ongoing human-driven environmental changes (Brady *et al.* 2019, and literature cited therein).

The outcomes of the interpopulation crossing further emphasize the crucial role played by the environmental contrasts in modulating the fitness responses of the different lineages. Our results showed that when aridity is introduced in the experimental system, interpopulation mating caused asymmetrical consequences in core versus marginal populations, with heterosis prevalent in the first and maladaptation prevalent in the latter. Then, incorporating future ecological scenarios in the analyses on fitness consequences of interpopulation mating indicated that the general assumption ‘larger is better’ does not always hold, because AGF from large core to small peripheral populations can undermine the advantage provided by local adaptation. On the other hand, the patterns of heterosis observed in our study evidenced that, in spite of its extreme genetic poorness (Gargano *et al.* 2015), the isolated marginal population is better adapted to cope with the expected effects of near-future CCs. Therefore, this population can be a potential source of genes for reducing maladaptation in the core unit under increasing aridity stress.

Various authors argued that peripheral populations could improve species responsiveness to climate-driven ecological variations, because they are adapted to unique environmental conditions (Rehm *et al.* 2015; Abeli *et al.* 2018). However, there is a lack of experimental evidence showing differences in the adaptive potential to CC components in ecological or geographical marginal plant populations. Dickman *et al.* (2019) recorded an enhanced adaptation to drought in seeds from marginal low-elevation populations of *M. laciniatus*; while Abeli *et al.* (2015) found latitude-driven tolerance to varying water availability between northern and peripheral southern populations of *S. suecica*. Furthermore, variations in seed tolerance to osmotic stress were found across the range of the crop wild relatives *Aegylops geniculata* and *Secale strictum* (Orsenigo *et al.* 2017; Rossi *et al.* 2020). The previous studies, however, did not experimentally test for adaptation to CC in small and isolated peripheral populations through interpopulation crossing. Based on our work, small and little viable populations occurring at the range margins can be an actual genetic source for improving the species adaptive potential against the ongoing CC, by reducing the extent of maladaptation that can rise under the novel ecological contexts on populations that are currently large and viable.

### Implications for designing actions of AGF under ongoing CC (Q4)

Our work provides some relevant insights about the role that marginal small populations could play in improving the conservation chances of rare plants under ongoing CC. Assisted gene flow is considered an effective way to improve genetic diversity and fitness of small and inbred demographic units (Aitken and Whitlock 2013; Frankham 2015; Whiteley *et al.* 2015). A previous study on *D. guliae* confirmed that, under neutral environment conditions, AGF might improve genetic richness and fitness of the southernmost imperilled demographic unit (Gargano *et al.* 2015). However, when environmental variations expected under CC are taken into account, the genetic rescue of the peripheral unit provided through AGF may be ineffective or even counterproductive. Under the increased aridity promoted by climate change, AGF – benefit may occur in the core population, thus working in the opposite direction than typically expected. The small border population can be the best donor candidate for reducing CC-driven maladaptation in the core units, which are the populations on which the species relies on for long-term persistence. Other authors pointed out that the evolutionary potential of plant species versus climate variations can be substantially enhanced by AGF among large population clines (Aitken and Bemmels 2016), marginal populations occurring in similar habitats (Morente-López *et al.* 2021) and from large to small border populations (Bontrager and Angert 2019). In this context, the present study highlights the importance of testing the effect of AGF under contrasting environments to set optimal AGF design under current and, especially, future ecological trajectories determined by ongoing global change. According to the predicted evolutionary values of marginal populations (Abeli *et al.* 2018), our work emphasizes the role played by small edge populations, typically representing the ‘recipient target’ of AGF, as a source of functional adaptation against CC.

Because patterns of ID and OD can vary across generations (Frankham 2015), extending fitness analyses beyond the F_1_ generation could provide a more robust picture of the consequences of the different pollination treatments, with a special focus on between-population crossing. Previous work on this species revealed that inbreeding induces severe and long-lasting fitness limitations (Gargano *et al.* 2011). On the contrary, no data are currently available on the long-term patterns of heterosis (or OD) that interpopulation gene flow can promote in subsequent generations. Such a question remained unaddressed in this paper, mainly due to the experimental limitations caused by the fitness limitations affecting the plants from the marginal population, which impeded to have a representative F_2_ sample. Relying on less rare species as study systems could help to overcome such drawbacks and to highlight findings of more general validity.

Finally, the fitness analyses presented in this study are prevalently based on estimations of female fitness components. Nonetheless, male fertility can also influence the reproductive success of animal-pollinated plants, often resulting in conflict with patterns of female fertility (e.g. Minnaar *et al.* 2019, and literature cited therein). In *D. guliae*, previous work revealed little signals of ID on pollen viability compared to female fertility traits (Gargano *et al.* 2011). However, no other components of male fitness were evaluated, as well as no information is available about the consequences of heterosis on the male fitness of this species. Future studies should address how AGF affects the different components of male fertility to give further insights on the actual benefit provided by AGF.

## Supporting Information

The following additional information is available in the online version of this article—

Appendix S1. Detailed protocol for plant cultivation.

Table S2.1. AICC scores for the different models carried out on binary (GERM, SURV) and continuous (ROOT, SHOOT) response variables. The asterisk indicates the model with best performance.

Table S2.2. Between-lineage fitness variations and related patterns of inbreeding depression and heterosis in the two populations for each trait and experimental treatment. Inbred (S), offspring obtained by self-pollination; Outbred (wC), offspring obtained by within-population cross-pollination; Hybrid (bC), offspring obtained by between-population cross-pollination; further details are provided in the main text.

Table S3.1. Data set relative to seed germinations.

Table S3.2. Radicle length and shoot size after 21 observation days.

Table S3.3. Survival data for 21 days old seedlings.

Figure S4.1. Mean germination rate recorded in each family under different pollination and stress protocols.

Figure S4.2. Mean radicle length recorded in each family under different pollination and stress treatments.

Figure S4.3. Mean shoot size recorded in each family under different pollination and stress treatments.

Figure S4.4. Mean survival rate recorded in each family under different pollination and stress treatments.

plac022_suppl_Supplementary_Appendix_S1

plac022_suppl_Supplementary_Appendix_S2

plac022_suppl_Supplementary_Appendix_S3

plac022_suppl_Supplementary_Appendix_S4

## Data Availability

All data supporting the findings of this study are available within its **Supporting Information**.

## Notes

**Natural History and Conservation. Chief Editor**: F. Xavier Picó
