# Supplementary material for: Do marginal plant populations enhance the fitness of larger core units under ongoing climate change? Empirical insights from a rare carnation
Source: AoB Plants. 2022 May 12;14(3):plac022. doi: 10.1093/aobpla/plac022 (PMC9167561; doi:10.1093/aobpla/plac022)
Supplement: plac022_suppl_Supplementary_Appendix_S1 [file plac022_suppl_supplementary_appendix_s1.pdf]

Appendix A1. Protocol of seed germination and cultivation of F_0 plants.

After a storage period of 60 days at 4 °C, the ripe seeds collected in the field were germinated on wet paper in Petri dishes at the Botanic Garden of the University of Calabria. The day following the appearance of the root apex, the seeds were transferred in 3.5 x 3.5 cm pots containing a mixture of peat moss and compost with presence of pumice fragments. Subsequently, 30-day old seedlings from the two populations were transplanted in cultivation beds filled with brown soil and subjected to periodical irrigation and fertilization with a mixture of ammonium (6 %), phosphorous (5 %), and potassium (7 %) diluted in H_2O. The plants were placed in common garden in a regular array (individuals were 20 cm apart), and kept apart from competing species throughout the whole experiment.
