# Supplementary material for: Do marginal plant populations enhance the fitness of larger core units under ongoing climate change? Empirical insights from a rare carnation
Source: AoB Plants. 2022 May 12;14(3):plac022. doi: 10.1093/aobpla/plac022 (PMC9167561; doi:10.1093/aobpla/plac022)
Supplement: plac022_suppl_Supplementary_Appendix_S2 [file plac022_suppl_supplementary_appendix_s2.pdf]

Appendix2. Additional tables

Table S2.1. AICC scores for the different models carried out on binary (GERM, SURV) and continuous (ROOT, SHOOT) response variables. The asterisk indicates the model with best performance.

Variable	Assumed distribution	Link-function	AICC	Selected model
GERM	Binomial	Complementary log-log	251.81	*
GERM	Binomial	Probit	253.47	
GERM	Binomial	Logit	254.21	
SURV	Binomial	Logit	210.04	*
SURV	Binomial	Probit	210.12	
SURV	Binomial	Complementary log-log	210.24	
ROOT	Normal	Log	2039.67	*
ROOT	Normal	Identity	2039.75	
SHOOT	Normal	Log	2184.33	*
SHOOT	Normal	Identity	2184.73	

Table S2.2. Between-lineage fitness variations and related patterns of inbreeding depression and heterosis in the two populations for each trait and experimental treatment. Inbred (S), offspring obtained by self-pollination; Outbred (wC), offspring obtained by within-population cross-pollination; Hybrid (bC), offspring obtained by between-population cross-pollination; further details are provided in the main text.

	Treatment	agar					wet					dry				
Population	Trait	Inbred (S)	Outbred (wC)	Hybrid (bC)	ID_{AGAR}	H	Inbred (S)	Outbred (wC)	Hybrid (bC)	ID_{WET}	H	Inbred (S)	Outbred (wC)	Hybrid (bC)	ID_{DRY}	H
Core	Seed germination (%)	0,85	1,00	0,90	0,15	-0,11	0,73	0,95	0,82	0,23	-0,16	0,67	0,71	0,90	0,06	0,21
Peripheral	Seed germination (%)	0,70	0,85	0,90	0,18	0,06	0,60	0,83	0,85	0,28	0,02	0,55	0,70	0,63	0,21	-0,11
Core	Early survival (%)	-	-	-	-	-	0,56	0,85	0,79	0,34	-0,08	0,54	0,68	0,77	0,21	0,12
Peripheral	Early survival (%)	-	-	-	-	-	0,60	0,70	0,83	0,14	0,16	0,48	0,62	0,45	0,23	-0,38
Core	Radicle length (mm)	-	-	-	-	-	15,55	23,94	18,41	0,35	-0,30	14,68	18,16	19,15	0,19	0,05
Peripheral	Radicle length (mm)	-	-	-	-	-	16,37	20,99	21,41	0,22	0,02	14,97	19,40	18,34	0,23	-0,06
Core	Shoot size (mm)	-	-	-	-	-	28,74	39,13	34,74	0,27	-0,13	29,07	34,28	38,04	0,15	0,10
Peripheral	Shoot size (mm)	-	-	-	-	-	30,31	37,19	39,67	0,18	0,06	29,18	37,08	33,49	0,21	-0,11
