# Supplementary material for: Do marginal plant populations enhance the fitness of larger core units under ongoing climate change? Empirical insights from a rare carnation
Source: AoB Plants. 2022 May 12;14(3):plac022. doi: 10.1093/aobpla/plac022 (PMC9167561; doi:10.1093/aobpla/plac022)
Supplement: plac022_suppl_Supplementary_Appendix_S3 [file plac022_suppl_supplementary_appendix_s3.docx]

**Appendix S3. Datasets relative to the fitness analyses described in the paper.**

**Table S3.1. Dataset relative to seed germinations.**

| Id_seed | Family | Population_type | Pollination_treatment | Stress_treatment | GERM[1,0] |
| --- | --- | --- | --- | --- | --- |
| 1 | Cor_1 | Core | between-population cross-pollination | agar | 1 |
| 2 | Cor_1 | Core | between-population cross-pollination | agar | 1 |
| 3 | Cor_1 | Core | between-population cross-pollination | agar | 1 |
| 4 | Cor_1 | Core | between-population cross-pollination | agar | 1 |
| 5 | Cor_1 | Core | between-population cross-pollination | dry | 1 |
| 6 | Cor_1 | Core | between-population cross-pollination | dry | 1 |
| 7 | Cor_1 | Core | between-population cross-pollination | dry | 1 |
| 8 | Cor_1 | Core | between-population cross-pollination | dry | 1 |
| 9 | Cor_1 | Core | between-population cross-pollination | dry | 1 |
| 10 | Cor_1 | Core | between-population cross-pollination | dry | 1 |
| 11 | Cor_1 | Core | between-population cross-pollination | dry | 1 |
| 12 | Cor_1 | Core | between-population cross-pollination | dry | 1 |
| 13 | Cor_1 | Core | between-population cross-pollination | wet | 0 |
| 14 | Cor_1 | Core | between-population cross-pollination | wet | 1 |
| 15 | Cor_1 | Core | between-population cross-pollination | wet | 1 |
| 16 | Cor_1 | Core | between-population cross-pollination | wet | 0 |
| 17 | Cor_1 | Core | between-population cross-pollination | wet | 1 |
| 18 | Cor_1 | Core | between-population cross-pollination | wet | 1 |
| 19 | Cor_1 | Core | between-population cross-pollination | wet | 1 |
| 20 | Cor_1 | Core | between-population cross-pollination | wet | 1 |
| 21 | Cor_1 | Core | within_population cross-pollination | agar | 1 |
| 22 | Cor_1 | Core | within_population cross-pollination | agar | 1 |
| 23 | Cor_1 | Core | within_population cross-pollination | agar | 1 |
| 24 | Cor_1 | Core | within_population cross-pollination | agar | 1 |
| 25 | Cor_1 | Core | within_population cross-pollination | dry | 1 |
| 26 | Cor_1 | Core | within_population cross-pollination | dry | 1 |
| 27 | Cor_1 | Core | within_population cross-pollination | dry | 1 |
| 28 | Cor_1 | Core | within_population cross-pollination | dry | 1 |
| 29 | Cor_1 | Core | within_population cross-pollination | dry | 1 |
| 30 | Cor_1 | Core | within_population cross-pollination | dry | 0 |
| 31 | Cor_1 | Core | within_population cross-pollination | dry | 1 |
| 32 | Cor_1 | Core | within_population cross-pollination | dry | 1 |
| 33 | Cor_1 | Core | within_population cross-pollination | wet | 1 |
| 34 | Cor_1 | Core | within_population cross-pollination | wet | 1 |
| 35 | Cor_1 | Core | within_population cross-pollination | wet | 1 |
| 36 | Cor_1 | Core | within_population cross-pollination | wet | 1 |
| 37 | Cor_1 | Core | within_population cross-pollination | wet | 1 |
| 38 | Cor_1 | Core | within_population cross-pollination | wet | 1 |
| 39 | Cor_1 | Core | within_population cross-pollination | wet | 1 |
| 40 | Cor_1 | Core | within_population cross-pollination | wet | 1 |
| 41 | Cor_1 | Core | self-pollination | agar | 1 |
| 42 | Cor_1 | Core | self-pollination | agar | 1 |
| 43 | Cor_1 | Core | self-pollination | agar | 1 |
| 44 | Cor_1 | Core | self-pollination | agar | 1 |
| 45 | Cor_1 | Core | self-pollination | dry | 1 |
| 46 | Cor_1 | Core | self-pollination | dry | 0 |
| 47 | Cor_1 | Core | self-pollination | dry | 1 |
| 48 | Cor_1 | Core | self-pollination | dry | 0 |
| 49 | Cor_1 | Core | self-pollination | dry | 1 |
| 50 | Cor_1 | Core | self-pollination | dry | 0 |
| 51 | Cor_1 | Core | self-pollination | dry | 1 |
| 52 | Cor_1 | Core | self-pollination | dry | 1 |
| 53 | Cor_1 | Core | self-pollination | wet | 0 |
| 54 | Cor_1 | Core | self-pollination | wet | 1 |
| 55 | Cor_1 | Core | self-pollination | wet | 1 |
| 56 | Cor_1 | Core | self-pollination | wet | 1 |
| 57 | Cor_1 | Core | self-pollination | wet | 0 |
| 58 | Cor_1 | Core | self-pollination | wet | 1 |
| 59 | Cor_1 | Core | self-pollination | wet | 1 |
| 60 | Cor_1 | Core | self-pollination | wet | 1 |
| 61 | Cor_2 | Core | between-population cross-pollination | agar | 1 |
| 62 | Cor_2 | Core | between-population cross-pollination | agar | 1 |
| 63 | Cor_2 | Core | between-population cross-pollination | agar | 1 |
| 64 | Cor_2 | Core | between-population cross-pollination | agar | 1 |
| 65 | Cor_2 | Core | between-population cross-pollination | dry | 1 |
| 66 | Cor_2 | Core | between-population cross-pollination | dry | 1 |
| 67 | Cor_2 | Core | between-population cross-pollination | dry | 1 |
| 68 | Cor_2 | Core | between-population cross-pollination | dry | 1 |
| 69 | Cor_2 | Core | between-population cross-pollination | dry | 1 |
| 70 | Cor_2 | Core | between-population cross-pollination | dry | 1 |
| 71 | Cor_2 | Core | between-population cross-pollination | dry | 1 |
| 72 | Cor_2 | Core | between-population cross-pollination | dry | 1 |
| 73 | Cor_2 | Core | between-population cross-pollination | wet | 1 |
| 74 | Cor_2 | Core | between-population cross-pollination | wet | 1 |
| 75 | Cor_2 | Core | between-population cross-pollination | wet | 1 |
| 76 | Cor_2 | Core | between-population cross-pollination | wet | 0 |
| 77 | Cor_2 | Core | between-population cross-pollination | wet | 1 |
| 78 | Cor_2 | Core | between-population cross-pollination | wet | 1 |
| 79 | Cor_2 | Core | between-population cross-pollination | wet | 1 |
| 80 | Cor_2 | Core | between-population cross-pollination | wet | 1 |
| 81 | Cor_2 | Core | within_population cross-pollination | agar | 1 |
| 82 | Cor_2 | Core | within_population cross-pollination | agar | 1 |
| 83 | Cor_2 | Core | within_population cross-pollination | agar | 1 |
| 84 | Cor_2 | Core | within_population cross-pollination | agar | 1 |
| 85 | Cor_2 | Core | within_population cross-pollination | dry | 1 |
| 86 | Cor_2 | Core | within_population cross-pollination | dry | 0 |
| 87 | Cor_2 | Core | within_population cross-pollination | dry | 1 |
| 88 | Cor_2 | Core | within_population cross-pollination | dry | 0 |
| 89 | Cor_2 | Core | within_population cross-pollination | dry | 1 |
| 90 | Cor_2 | Core | within_population cross-pollination | dry | 1 |
| 91 | Cor_2 | Core | within_population cross-pollination | dry | 0 |
| 92 | Cor_2 | Core | within_population cross-pollination | dry | 1 |
| 93 | Cor_2 | Core | within_population cross-pollination | wet | 1 |
| 94 | Cor_2 | Core | within_population cross-pollination | wet | 1 |
| 95 | Cor_2 | Core | within_population cross-pollination | wet | 1 |
| 96 | Cor_2 | Core | within_population cross-pollination | wet | 1 |
| 97 | Cor_2 | Core | within_population cross-pollination | wet | 1 |
| 98 | Cor_2 | Core | within_population cross-pollination | wet | 1 |
| 99 | Cor_2 | Core | within_population cross-pollination | wet | 1 |
| 100 | Cor_2 | Core | within_population cross-pollination | wet | 1 |
| 101 | Cor_2 | Core | self-pollination | agar | 1 |
| 102 | Cor_2 | Core | self-pollination | agar | 0 |
| 103 | Cor_2 | Core | self-pollination | agar | 1 |
| 104 | Cor_2 | Core | self-pollination | agar | 1 |
| 105 | Cor_2 | Core | self-pollination | dry | 1 |
| 106 | Cor_2 | Core | self-pollination | dry | 1 |
| 107 | Cor_2 | Core | self-pollination | dry | 1 |
| 108 | Cor_2 | Core | self-pollination | dry | 0 |
| 109 | Cor_2 | Core | self-pollination | dry | 1 |
| 110 | Cor_2 | Core | self-pollination | dry | 1 |
| 111 | Cor_2 | Core | self-pollination | dry | 0 |
| 112 | Cor_2 | Core | self-pollination | dry | 0 |
| 113 | Cor_2 | Core | self-pollination | wet | 1 |
| 114 | Cor_2 | Core | self-pollination | wet | 1 |
| 115 | Cor_2 | Core | self-pollination | wet | 1 |
| 116 | Cor_2 | Core | self-pollination | wet | 1 |
| 117 | Cor_2 | Core | self-pollination | wet | 1 |
| 118 | Cor_2 | Core | self-pollination | wet | 1 |
| 119 | Cor_2 | Core | self-pollination | wet | 0 |
| 120 | Cor_2 | Core | self-pollination | wet | 1 |
| 121 | Cor_2 | Core | self-pollination | wet | 1 |
| 122 | Cor_3 | Core | between-population cross-pollination | agar | 1 |
| 123 | Cor_3 | Core | between-population cross-pollination | agar | 1 |
| 124 | Cor_3 | Core | between-population cross-pollination | agar | 1 |
| 125 | Cor_3 | Core | between-population cross-pollination | agar | 1 |
| 126 | Cor_3 | Core | between-population cross-pollination | dry | 1 |
| 127 | Cor_3 | Core | between-population cross-pollination | dry | 1 |
| 128 | Cor_3 | Core | between-population cross-pollination | dry | 1 |
| 129 | Cor_3 | Core | between-population cross-pollination | dry | 1 |
| 130 | Cor_3 | Core | between-population cross-pollination | dry | 1 |
| 131 | Cor_3 | Core | between-population cross-pollination | dry | 1 |
| 132 | Cor_3 | Core | between-population cross-pollination | dry | 1 |
| 133 | Cor_3 | Core | between-population cross-pollination | dry | 1 |
| 134 | Cor_3 | Core | between-population cross-pollination | wet | 1 |
| 135 | Cor_3 | Core | between-population cross-pollination | wet | 1 |
| 136 | Cor_3 | Core | between-population cross-pollination | wet | 1 |
| 137 | Cor_3 | Core | between-population cross-pollination | wet | 0 |
| 138 | Cor_3 | Core | between-population cross-pollination | wet | 1 |
| 139 | Cor_3 | Core | between-population cross-pollination | wet | 1 |
| 140 | Cor_3 | Core | between-population cross-pollination | wet | 1 |
| 141 | Cor_3 | Core | between-population cross-pollination | wet | 0 |
| 142 | Cor_3 | Core | within_population cross-pollination | agar | 1 |
| 143 | Cor_3 | Core | within_population cross-pollination | agar | 1 |
| 144 | Cor_3 | Core | within_population cross-pollination | agar | 1 |
| 145 | Cor_3 | Core | within_population cross-pollination | agar | 1 |
| 146 | Cor_3 | Core | within_population cross-pollination | dry | 1 |
| 147 | Cor_3 | Core | within_population cross-pollination | dry | 1 |
| 148 | Cor_3 | Core | within_population cross-pollination | dry | 1 |
| 149 | Cor_3 | Core | within_population cross-pollination | dry | 1 |
| 150 | Cor_3 | Core | within_population cross-pollination | dry | 1 |
| 151 | Cor_3 | Core | within_population cross-pollination | dry | 1 |
| 152 | Cor_3 | Core | within_population cross-pollination | dry | 0 |
| 153 | Cor_3 | Core | within_population cross-pollination | dry | 1 |
| 154 | Cor_3 | Core | within_population cross-pollination | dry | 0 |
| 155 | Cor_3 | Core | within_population cross-pollination | wet | 1 |
| 156 | Cor_3 | Core | within_population cross-pollination | wet | 1 |
| 157 | Cor_3 | Core | within_population cross-pollination | wet | 1 |
| 158 | Cor_3 | Core | within_population cross-pollination | wet | 1 |
| 159 | Cor_3 | Core | within_population cross-pollination | wet | 0 |
| 160 | Cor_3 | Core | within_population cross-pollination | wet | 1 |
| 161 | Cor_3 | Core | within_population cross-pollination | wet | 1 |
| 162 | Cor_3 | Core | within_population cross-pollination | wet | 1 |
| 163 | Cor_3 | Core | self-pollination | agar | 0 |
| 164 | Cor_3 | Core | self-pollination | agar | 1 |
| 165 | Cor_3 | Core | self-pollination | agar | 1 |
| 166 | Cor_3 | Core | self-pollination | agar | 0 |
| 167 | Cor_3 | Core | self-pollination | dry | 1 |
| 168 | Cor_3 | Core | self-pollination | dry | 1 |
| 169 | Cor_3 | Core | self-pollination | dry | 0 |
| 170 | Cor_3 | Core | self-pollination | dry | 1 |
| 171 | Cor_3 | Core | self-pollination | dry | 1 |
| 172 | Cor_3 | Core | self-pollination | dry | 1 |
| 173 | Cor_3 | Core | self-pollination | dry | 0 |
| 174 | Cor_3 | Core | self-pollination | dry | 0 |
| 175 | Cor_3 | Core | self-pollination | wet | 0 |
| 176 | Cor_3 | Core | self-pollination | wet | 1 |
| 177 | Cor_3 | Core | self-pollination | wet | 1 |
| 178 | Cor_3 | Core | self-pollination | wet | 0 |
| 179 | Cor_3 | Core | self-pollination | wet | 1 |
| 180 | Cor_3 | Core | self-pollination | wet | 1 |
| 181 | Cor_3 | Core | self-pollination | wet | 1 |
| 182 | Cor_3 | Core | self-pollination | wet | 0 |
| 183 | Cor_4 | Core | between-population cross-pollination | agar | 1 |
| 184 | Cor_4 | Core | between-population cross-pollination | agar | 0 |
| 185 | Cor_4 | Core | between-population cross-pollination | agar | 1 |
| 186 | Cor_4 | Core | between-population cross-pollination | agar | 1 |
| 187 | Cor_4 | Core | between-population cross-pollination | dry | 1 |
| 188 | Cor_4 | Core | between-population cross-pollination | dry | 1 |
| 189 | Cor_4 | Core | between-population cross-pollination | dry | 1 |
| 190 | Cor_4 | Core | between-population cross-pollination | dry | 1 |
| 191 | Cor_4 | Core | between-population cross-pollination | dry | 1 |
| 192 | Cor_4 | Core | between-population cross-pollination | dry | 1 |
| 193 | Cor_4 | Core | between-population cross-pollination | dry | 1 |
| 194 | Cor_4 | Core | between-population cross-pollination | dry | 1 |
| 195 | Cor_4 | Core | between-population cross-pollination | wet | 1 |
| 196 | Cor_4 | Core | between-population cross-pollination | wet | 1 |
| 197 | Cor_4 | Core | between-population cross-pollination | wet | 0 |
| 198 | Cor_4 | Core | between-population cross-pollination | wet | 1 |
| 199 | Cor_4 | Core | between-population cross-pollination | wet | 1 |
| 200 | Cor_4 | Core | between-population cross-pollination | wet | 1 |
| 201 | Cor_4 | Core | between-population cross-pollination | wet | 1 |
| 202 | Cor_4 | Core | between-population cross-pollination | wet | 1 |
| 203 | Cor_4 | Core | within_population cross-pollination | agar | 1 |
| 204 | Cor_4 | Core | within_population cross-pollination | agar | 1 |
| 205 | Cor_4 | Core | within_population cross-pollination | agar | 1 |
| 206 | Cor_4 | Core | within_population cross-pollination | agar | 1 |
| 207 | Cor_4 | Core | within_population cross-pollination | dry | 1 |
| 208 | Cor_4 | Core | within_population cross-pollination | dry | 0 |
| 209 | Cor_4 | Core | within_population cross-pollination | dry | 1 |
| 210 | Cor_4 | Core | within_population cross-pollination | dry | 1 |
| 211 | Cor_4 | Core | within_population cross-pollination | dry | 0 |
| 212 | Cor_4 | Core | within_population cross-pollination | dry | 1 |
| 213 | Cor_4 | Core | within_population cross-pollination | dry | 0 |
| 214 | Cor_4 | Core | within_population cross-pollination | dry | 1 |
| 215 | Cor_4 | Core | within_population cross-pollination | wet | 1 |
| 216 | Cor_4 | Core | within_population cross-pollination | wet | 1 |
| 217 | Cor_4 | Core | within_population cross-pollination | wet | 1 |
| 218 | Cor_4 | Core | within_population cross-pollination | wet | 1 |
| 219 | Cor_4 | Core | within_population cross-pollination | wet | 1 |
| 220 | Cor_4 | Core | within_population cross-pollination | wet | 1 |
| 221 | Cor_4 | Core | within_population cross-pollination | wet | 1 |
| 222 | Cor_4 | Core | within_population cross-pollination | wet | 1 |
| 223 | Cor_4 | Core | self-pollination | agar | 1 |
| 224 | Cor_4 | Core | self-pollination | agar | 1 |
| 225 | Cor_4 | Core | self-pollination | agar | 1 |
| 226 | Cor_4 | Core | self-pollination | agar | 1 |
| 227 | Cor_4 | Core | self-pollination | dry | 1 |
| 228 | Cor_4 | Core | self-pollination | dry | 1 |
| 229 | Cor_4 | Core | self-pollination | dry | 1 |
| 230 | Cor_4 | Core | self-pollination | dry | 0 |
| 231 | Cor_4 | Core | self-pollination | dry | 1 |
| 232 | Cor_4 | Core | self-pollination | dry | 1 |
| 233 | Cor_4 | Core | self-pollination | dry | 0 |
| 234 | Cor_4 | Core | self-pollination | dry | 0 |
| 235 | Cor_4 | Core | self-pollination | wet | 0 |
| 236 | Cor_4 | Core | self-pollination | wet | 1 |
| 237 | Cor_4 | Core | self-pollination | wet | 1 |
| 238 | Cor_4 | Core | self-pollination | wet | 0 |
| 239 | Cor_4 | Core | self-pollination | wet | 1 |
| 240 | Cor_4 | Core | self-pollination | wet | 1 |
| 241 | Cor_4 | Core | self-pollination | wet | 1 |
| 242 | Cor_4 | Core | self-pollination | wet | 0 |
| 243 | Cor_5 | Core | between-population cross-pollination | agar | 1 |
| 244 | Cor_5 | Core | between-population cross-pollination | agar | 0 |
| 245 | Cor_5 | Core | between-population cross-pollination | agar | 1 |
| 246 | Cor_5 | Core | between-population cross-pollination | agar | 1 |
| 247 | Cor_5 | Core | between-population cross-pollination | agar | 1 |
| 248 | Cor_5 | Core | between-population cross-pollination | dry | 0 |
| 249 | Cor_5 | Core | between-population cross-pollination | dry | 1 |
| 250 | Cor_5 | Core | between-population cross-pollination | dry | 0 |
| 251 | Cor_5 | Core | between-population cross-pollination | dry | 0 |
| 252 | Cor_5 | Core | between-population cross-pollination | dry | 1 |
| 253 | Cor_5 | Core | between-population cross-pollination | dry | 1 |
| 254 | Cor_5 | Core | between-population cross-pollination | dry | 1 |
| 255 | Cor_5 | Core | between-population cross-pollination | dry | 0 |
| 256 | Cor_5 | Core | between-population cross-pollination | wet | 1 |
| 257 | Cor_5 | Core | between-population cross-pollination | wet | 1 |
| 258 | Cor_5 | Core | between-population cross-pollination | wet | 0 |
| 259 | Cor_5 | Core | between-population cross-pollination | wet | 1 |
| 260 | Cor_5 | Core | between-population cross-pollination | wet | 1 |
| 261 | Cor_5 | Core | between-population cross-pollination | wet | 1 |
| 262 | Cor_5 | Core | between-population cross-pollination | wet | 1 |
| 263 | Cor_5 | Core | within_population cross-pollination | agar | 1 |
| 264 | Cor_5 | Core | within_population cross-pollination | agar | 1 |
| 265 | Cor_5 | Core | within_population cross-pollination | agar | 1 |
| 266 | Cor_5 | Core | within_population cross-pollination | dry | 1 |
| 267 | Cor_5 | Core | within_population cross-pollination | dry | 0 |
| 268 | Cor_5 | Core | within_population cross-pollination | dry | 1 |
| 269 | Cor_5 | Core | within_population cross-pollination | dry | 0 |
| 270 | Cor_5 | Core | within_population cross-pollination | dry | 0 |
| 271 | Cor_5 | Core | within_population cross-pollination | dry | 1 |
| 272 | Cor_5 | Core | within_population cross-pollination | dry | 1 |
| 273 | Cor_5 | Core | within_population cross-pollination | dry | 1 |
| 274 | Cor_5 | Core | within_population cross-pollination | wet | 1 |
| 275 | Cor_5 | Core | within_population cross-pollination | wet | 1 |
| 276 | Cor_5 | Core | within_population cross-pollination | wet | 1 |
| 277 | Cor_5 | Core | within_population cross-pollination | wet | 0 |
| 278 | Cor_5 | Core | within_population cross-pollination | wet | 1 |
| 279 | Cor_5 | Core | within_population cross-pollination | wet | 1 |
| 280 | Cor_5 | Core | within_population cross-pollination | wet | 1 |
| 281 | Cor_5 | Core | within_population cross-pollination | wet | 1 |
| 282 | Cor_5 | Core | self-pollination | agar | 1 |
| 283 | Cor_5 | Core | self-pollination | agar | 1 |
| 284 | Cor_5 | Core | self-pollination | agar | 1 |
| 285 | Cor_5 | Core | self-pollination | agar | 1 |
| 286 | Cor_5 | Core | self-pollination | dry | 1 |
| 287 | Cor_5 | Core | self-pollination | dry | 1 |
| 288 | Cor_5 | Core | self-pollination | dry | 1 |
| 289 | Cor_5 | Core | self-pollination | dry | 1 |
| 290 | Cor_5 | Core | self-pollination | dry | 1 |
| 291 | Cor_5 | Core | self-pollination | dry | 0 |
| 292 | Cor_5 | Core | self-pollination | dry | 1 |
| 293 | Cor_5 | Core | self-pollination | wet | 1 |
| 294 | Cor_5 | Core | self-pollination | wet | 1 |
| 295 | Cor_5 | Core | self-pollination | wet | 0 |
| 296 | Cor_5 | Core | self-pollination | wet | 1 |
| 297 | Cor_5 | Core | self-pollination | wet | 1 |
| 298 | Cor_5 | Core | self-pollination | wet | 1 |
| 299 | Cor_5 | Core | self-pollination | wet | 1 |
| 300 | Cor_5 | Core | self-pollination | wet | 0 |
| 301 | Per_1 | Peripheral | between-population cross-pollination | agar | 1 |
| 302 | Per_1 | Peripheral | between-population cross-pollination | agar | 1 |
| 303 | Per_1 | Peripheral | between-population cross-pollination | agar | 0 |
| 304 | Per_1 | Peripheral | between-population cross-pollination | agar | 1 |
| 305 | Per_1 | Peripheral | between-population cross-pollination | agar | 1 |
| 306 | Per_1 | Peripheral | between-population cross-pollination | agar | 1 |
| 307 | Per_1 | Peripheral | between-population cross-pollination | agar | 1 |
| 308 | Per_1 | Peripheral | between-population cross-pollination | agar | 1 |
| 309 | Per_1 | Peripheral | between-population cross-pollination | dry | 0 |
| 310 | Per_1 | Peripheral | between-population cross-pollination | dry | 0 |
| 311 | Per_1 | Peripheral | between-population cross-pollination | dry | 1 |
| 312 | Per_1 | Peripheral | between-population cross-pollination | dry | 1 |
| 313 | Per_1 | Peripheral | between-population cross-pollination | dry | 0 |
| 314 | Per_1 | Peripheral | between-population cross-pollination | dry | 0 |
| 315 | Per_1 | Peripheral | between-population cross-pollination | dry | 1 |
| 316 | Per_1 | Peripheral | between-population cross-pollination | dry | 1 |
| 317 | Per_1 | Peripheral | between-population cross-pollination | wet | 1 |
| 318 | Per_1 | Peripheral | between-population cross-pollination | wet | 1 |
| 319 | Per_1 | Peripheral | between-population cross-pollination | wet | 1 |
| 320 | Per_1 | Peripheral | between-population cross-pollination | wet | 0 |
| 321 | Per_1 | Peripheral | within_population cross-pollination | agar | 0 |
| 322 | Per_1 | Peripheral | within_population cross-pollination | agar | 1 |
| 323 | Per_1 | Peripheral | within_population cross-pollination | agar | 1 |
| 324 | Per_1 | Peripheral | within_population cross-pollination | agar | 1 |
| 325 | Per_1 | Peripheral | within_population cross-pollination | dry | 0 |
| 326 | Per_1 | Peripheral | within_population cross-pollination | dry | 1 |
| 327 | Per_1 | Peripheral | within_population cross-pollination | dry | 0 |
| 328 | Per_1 | Peripheral | within_population cross-pollination | dry | 0 |
| 329 | Per_1 | Peripheral | within_population cross-pollination | dry | 1 |
| 330 | Per_1 | Peripheral | within_population cross-pollination | dry | 1 |
| 331 | Per_1 | Peripheral | within_population cross-pollination | dry | 1 |
| 332 | Per_1 | Peripheral | within_population cross-pollination | dry | 1 |
| 333 | Per_1 | Peripheral | within_population cross-pollination | wet | 1 |
| 334 | Per_1 | Peripheral | within_population cross-pollination | wet | 1 |
| 335 | Per_1 | Peripheral | within_population cross-pollination | wet | 1 |
| 336 | Per_1 | Peripheral | within_population cross-pollination | wet | 1 |
| 337 | Per_1 | Peripheral | within_population cross-pollination | wet | 0 |
| 338 | Per_1 | Peripheral | within_population cross-pollination | wet | 1 |
| 339 | Per_1 | Peripheral | within_population cross-pollination | wet | 1 |
| 340 | Per_1 | Peripheral | within_population cross-pollination | wet | 1 |
| 341 | Per_1 | Peripheral | self-pollination | agar | 1 |
| 342 | Per_1 | Peripheral | self-pollination | agar | 0 |
| 343 | Per_1 | Peripheral | self-pollination | agar | 1 |
| 344 | Per_1 | Peripheral | self-pollination | agar | 0 |
| 345 | Per_1 | Peripheral | self-pollination | agar | 0 |
| 346 | Per_1 | Peripheral | self-pollination | agar | 1 |
| 347 | Per_1 | Peripheral | self-pollination | agar | 1 |
| 348 | Per_1 | Peripheral | self-pollination | agar | 0 |
| 349 | Per_1 | Peripheral | self-pollination | dry | 0 |
| 350 | Per_1 | Peripheral | self-pollination | dry | 1 |
| 351 | Per_1 | Peripheral | self-pollination | dry | 1 |
| 352 | Per_1 | Peripheral | self-pollination | dry | 1 |
| 353 | Per_1 | Peripheral | self-pollination | wet | 1 |
| 354 | Per_1 | Peripheral | self-pollination | wet | 0 |
| 355 | Per_1 | Peripheral | self-pollination | wet | 0 |
| 356 | Per_1 | Peripheral | self-pollination | wet | 1 |
| 357 | Per_1 | Peripheral | self-pollination | wet | 1 |
| 358 | Per_1 | Peripheral | self-pollination | wet | 1 |
| 359 | Per_1 | Peripheral | self-pollination | wet | 1 |
| 360 | Per_1 | Peripheral | self-pollination | wet | 0 |
| 361 | Per_2 | Peripheral | between-population cross-pollination | agar | 1 |
| 362 | Per_2 | Peripheral | between-population cross-pollination | agar | 0 |
| 363 | Per_2 | Peripheral | between-population cross-pollination | agar | 1 |
| 364 | Per_2 | Peripheral | between-population cross-pollination | agar | 1 |
| 365 | Per_2 | Peripheral | between-population cross-pollination | agar | 1 |
| 366 | Per_2 | Peripheral | between-population cross-pollination | agar | 1 |
| 367 | Per_2 | Peripheral | between-population cross-pollination | agar | 1 |
| 368 | Per_2 | Peripheral | between-population cross-pollination | agar | 1 |
| 369 | Per_2 | Peripheral | between-population cross-pollination | dry | 1 |
| 370 | Per_2 | Peripheral | between-population cross-pollination | dry | 0 |
| 371 | Per_2 | Peripheral | between-population cross-pollination | dry | 1 |
| 372 | Per_2 | Peripheral | between-population cross-pollination | dry | 0 |
| 373 | Per_2 | Peripheral | between-population cross-pollination | dry | 1 |
| 374 | Per_2 | Peripheral | between-population cross-pollination | dry | 1 |
| 375 | Per_2 | Peripheral | between-population cross-pollination | dry | 1 |
| 376 | Per_2 | Peripheral | between-population cross-pollination | dry | 0 |
| 377 | Per_2 | Peripheral | between-population cross-pollination | wet | 1 |
| 378 | Per_2 | Peripheral | between-population cross-pollination | wet | 0 |
| 379 | Per_2 | Peripheral | between-population cross-pollination | wet | 1 |
| 380 | Per_2 | Peripheral | between-population cross-pollination | wet | 1 |
| 381 | Per_2 | Peripheral | within_population cross-pollination | agar | 1 |
| 382 | Per_2 | Peripheral | within_population cross-pollination | agar | 1 |
| 383 | Per_2 | Peripheral | within_population cross-pollination | agar | 1 |
| 384 | Per_2 | Peripheral | within_population cross-pollination | agar | 1 |
| 385 | Per_2 | Peripheral | within_population cross-pollination | dry | 1 |
| 386 | Per_2 | Peripheral | within_population cross-pollination | dry | 1 |
| 387 | Per_2 | Peripheral | within_population cross-pollination | dry | 0 |
| 388 | Per_2 | Peripheral | within_population cross-pollination | dry | 1 |
| 389 | Per_2 | Peripheral | within_population cross-pollination | dry | 0 |
| 390 | Per_2 | Peripheral | within_population cross-pollination | dry | 1 |
| 391 | Per_2 | Peripheral | within_population cross-pollination | dry | 1 |
| 392 | Per_2 | Peripheral | within_population cross-pollination | dry | 1 |
| 393 | Per_2 | Peripheral | within_population cross-pollination | wet | 0 |
| 394 | Per_2 | Peripheral | within_population cross-pollination | wet | 1 |
| 395 | Per_2 | Peripheral | within_population cross-pollination | wet | 0 |
| 396 | Per_2 | Peripheral | within_population cross-pollination | wet | 0 |
| 397 | Per_2 | Peripheral | within_population cross-pollination | wet | 1 |
| 398 | Per_2 | Peripheral | within_population cross-pollination | wet | 1 |
| 399 | Per_2 | Peripheral | within_population cross-pollination | wet | 1 |
| 400 | Per_2 | Peripheral | within_population cross-pollination | wet | 1 |
| 401 | Per_2 | Peripheral | self-pollination | agar | 1 |
| 402 | Per_2 | Peripheral | self-pollination | agar | 0 |
| 403 | Per_2 | Peripheral | self-pollination | agar | 0 |
| 404 | Per_2 | Peripheral | self-pollination | agar | 1 |
| 405 | Per_2 | Peripheral | self-pollination | agar | 1 |
| 406 | Per_2 | Peripheral | self-pollination | agar | 1 |
| 407 | Per_2 | Peripheral | self-pollination | agar | 1 |
| 408 | Per_2 | Peripheral | self-pollination | agar | 1 |
| 409 | Per_2 | Peripheral | self-pollination | dry | 0 |
| 410 | Per_2 | Peripheral | self-pollination | dry | 0 |
| 411 | Per_2 | Peripheral | self-pollination | dry | 1 |
| 412 | Per_2 | Peripheral | self-pollination | dry | 1 |
| 413 | Per_2 | Peripheral | self-pollination | wet | 1 |
| 414 | Per_2 | Peripheral | self-pollination | wet | 1 |
| 415 | Per_2 | Peripheral | self-pollination | wet | 1 |
| 416 | Per_2 | Peripheral | self-pollination | wet | 0 |
| 417 | Per_2 | Peripheral | self-pollination | wet | 1 |
| 418 | Per_2 | Peripheral | self-pollination | wet | 1 |
| 419 | Per_2 | Peripheral | self-pollination | wet | 1 |
| 420 | Per_2 | Peripheral | self-pollination | wet | 0 |
| 421 | Per_3 | Peripheral | between-population cross-pollination | agar | 1 |
| 422 | Per_3 | Peripheral | between-population cross-pollination | agar | 0 |
| 423 | Per_3 | Peripheral | between-population cross-pollination | agar | 1 |
| 424 | Per_3 | Peripheral | between-population cross-pollination | agar | 1 |
| 425 | Per_3 | Peripheral | between-population cross-pollination | agar | 1 |
| 426 | Per_3 | Peripheral | between-population cross-pollination | agar | 1 |
| 427 | Per_3 | Peripheral | between-population cross-pollination | agar | 1 |
| 428 | Per_3 | Peripheral | between-population cross-pollination | agar | 1 |
| 429 | Per_3 | Peripheral | between-population cross-pollination | dry | 1 |
| 430 | Per_3 | Peripheral | between-population cross-pollination | dry | 1 |
| 431 | Per_3 | Peripheral | between-population cross-pollination | dry | 0 |
| 432 | Per_3 | Peripheral | between-population cross-pollination | dry | 0 |
| 433 | Per_3 | Peripheral | between-population cross-pollination | dry | 1 |
| 434 | Per_3 | Peripheral | between-population cross-pollination | dry | 1 |
| 435 | Per_3 | Peripheral | between-population cross-pollination | dry | 1 |
| 436 | Per_3 | Peripheral | between-population cross-pollination | dry | 0 |
| 437 | Per_3 | Peripheral | between-population cross-pollination | wet | 1 |
| 438 | Per_3 | Peripheral | between-population cross-pollination | wet | 1 |
| 439 | Per_3 | Peripheral | between-population cross-pollination | wet | 0 |
| 440 | Per_3 | Peripheral | between-population cross-pollination | wet | 1 |
| 441 | Per_3 | Peripheral | within_population cross-pollination | agar | 0 |
| 442 | Per_3 | Peripheral | within_population cross-pollination | agar | 1 |
| 443 | Per_3 | Peripheral | within_population cross-pollination | agar | 1 |
| 444 | Per_3 | Peripheral | within_population cross-pollination | agar | 1 |
| 445 | Per_3 | Peripheral | within_population cross-pollination | dry | 1 |
| 446 | Per_3 | Peripheral | within_population cross-pollination | dry | 0 |
| 447 | Per_3 | Peripheral | within_population cross-pollination | dry | 0 |
| 448 | Per_3 | Peripheral | within_population cross-pollination | dry | 1 |
| 449 | Per_3 | Peripheral | within_population cross-pollination | dry | 1 |
| 450 | Per_3 | Peripheral | within_population cross-pollination | dry | 1 |
| 451 | Per_3 | Peripheral | within_population cross-pollination | dry | 1 |
| 452 | Per_3 | Peripheral | within_population cross-pollination | dry | 1 |
| 453 | Per_3 | Peripheral | within_population cross-pollination | wet | 1 |
| 454 | Per_3 | Peripheral | within_population cross-pollination | wet | 0 |
| 455 | Per_3 | Peripheral | within_population cross-pollination | wet | 0 |
| 456 | Per_3 | Peripheral | within_population cross-pollination | wet | 1 |
| 457 | Per_3 | Peripheral | within_population cross-pollination | wet | 1 |
| 458 | Per_3 | Peripheral | within_population cross-pollination | wet | 1 |
| 459 | Per_3 | Peripheral | within_population cross-pollination | wet | 1 |
| 460 | Per_3 | Peripheral | within_population cross-pollination | wet | 1 |
| 461 | Per_3 | Peripheral | self-pollination | agar | 0 |
| 462 | Per_3 | Peripheral | self-pollination | agar | 0 |
| 463 | Per_3 | Peripheral | self-pollination | agar | 0 |
| 464 | Per_3 | Peripheral | self-pollination | agar | 1 |
| 465 | Per_3 | Peripheral | self-pollination | agar | 1 |
| 466 | Per_3 | Peripheral | self-pollination | agar | 1 |
| 467 | Per_3 | Peripheral | self-pollination | agar | 1 |
| 468 | Per_3 | Peripheral | self-pollination | agar | 1 |
| 469 | Per_3 | Peripheral | self-pollination | dry | 1 |
| 470 | Per_3 | Peripheral | self-pollination | dry | 0 |
| 471 | Per_3 | Peripheral | self-pollination | dry | 1 |
| 472 | Per_3 | Peripheral | self-pollination | dry | 1 |
| 473 | Per_3 | Peripheral | self-pollination | wet | 1 |
| 474 | Per_3 | Peripheral | self-pollination | wet | 1 |
| 475 | Per_3 | Peripheral | self-pollination | wet | 0 |
| 476 | Per_3 | Peripheral | self-pollination | wet | 0 |
| 477 | Per_3 | Peripheral | self-pollination | wet | 0 |
| 478 | Per_3 | Peripheral | self-pollination | wet | 1 |
| 479 | Per_3 | Peripheral | self-pollination | wet | 1 |
| 480 | Per_3 | Peripheral | self-pollination | wet | 0 |
| 481 | Per_4 | Peripheral | between-population cross-pollination | agar | 1 |
| 482 | Per_4 | Peripheral | between-population cross-pollination | agar | 1 |
| 483 | Per_4 | Peripheral | between-population cross-pollination | agar | 1 |
| 484 | Per_4 | Peripheral | between-population cross-pollination | agar | 1 |
| 485 | Per_4 | Peripheral | between-population cross-pollination | agar | 1 |
| 486 | Per_4 | Peripheral | between-population cross-pollination | agar | 1 |
| 487 | Per_4 | Peripheral | between-population cross-pollination | agar | 1 |
| 488 | Per_4 | Peripheral | between-population cross-pollination | agar | 1 |
| 489 | Per_4 | Peripheral | between-population cross-pollination | dry | 0 |
| 490 | Per_4 | Peripheral | between-population cross-pollination | dry | 1 |
| 491 | Per_4 | Peripheral | between-population cross-pollination | dry | 0 |
| 492 | Per_4 | Peripheral | between-population cross-pollination | dry | 1 |
| 493 | Per_4 | Peripheral | between-population cross-pollination | dry | 1 |
| 494 | Per_4 | Peripheral | between-population cross-pollination | dry | 1 |
| 495 | Per_4 | Peripheral | between-population cross-pollination | dry | 1 |
| 496 | Per_4 | Peripheral | between-population cross-pollination | dry | 1 |
| 497 | Per_4 | Peripheral | between-population cross-pollination | wet | 1 |
| 498 | Per_4 | Peripheral | between-population cross-pollination | wet | 1 |
| 499 | Per_4 | Peripheral | between-population cross-pollination | wet | 1 |
| 500 | Per_4 | Peripheral | between-population cross-pollination | wet | 1 |
| 501 | Per_4 | Peripheral | within_population cross-pollination | agar | 0 |
| 502 | Per_4 | Peripheral | within_population cross-pollination | agar | 1 |
| 503 | Per_4 | Peripheral | within_population cross-pollination | agar | 1 |
| 504 | Per_4 | Peripheral | within_population cross-pollination | agar | 1 |
| 505 | Per_4 | Peripheral | within_population cross-pollination | dry | 1 |
| 506 | Per_4 | Peripheral | within_population cross-pollination | dry | 1 |
| 507 | Per_4 | Peripheral | within_population cross-pollination | dry | 0 |
| 508 | Per_4 | Peripheral | within_population cross-pollination | dry | 1 |
| 509 | Per_4 | Peripheral | within_population cross-pollination | dry | 0 |
| 510 | Per_4 | Peripheral | within_population cross-pollination | dry | 1 |
| 511 | Per_4 | Peripheral | within_population cross-pollination | dry | 1 |
| 512 | Per_4 | Peripheral | within_population cross-pollination | dry | 0 |
| 513 | Per_4 | Peripheral | within_population cross-pollination | wet | 1 |
| 514 | Per_4 | Peripheral | within_population cross-pollination | wet | 1 |
| 515 | Per_4 | Peripheral | within_population cross-pollination | wet | 1 |
| 516 | Per_4 | Peripheral | within_population cross-pollination | wet | 1 |
| 517 | Per_4 | Peripheral | within_population cross-pollination | wet | 1 |
| 518 | Per_4 | Peripheral | within_population cross-pollination | wet | 1 |
| 519 | Per_4 | Peripheral | within_population cross-pollination | wet | 1 |
| 520 | Per_4 | Peripheral | within_population cross-pollination | wet | 1 |
| 521 | Per_4 | Peripheral | self-pollination | agar | 0 |
| 522 | Per_4 | Peripheral | self-pollination | agar | 1 |
| 523 | Per_4 | Peripheral | self-pollination | agar | 0 |
| 524 | Per_4 | Peripheral | self-pollination | agar | 1 |
| 525 | Per_4 | Peripheral | self-pollination | agar | 1 |
| 526 | Per_4 | Peripheral | self-pollination | agar | 1 |
| 527 | Per_4 | Peripheral | self-pollination | agar | 1 |
| 528 | Per_4 | Peripheral | self-pollination | agar | 1 |
| 529 | Per_4 | Peripheral | self-pollination | dry | 0 |
| 530 | Per_4 | Peripheral | self-pollination | dry | 0 |
| 531 | Per_4 | Peripheral | self-pollination | dry | 1 |
| 532 | Per_4 | Peripheral | self-pollination | dry | 0 |
| 533 | Per_4 | Peripheral | self-pollination | wet | 1 |
| 534 | Per_4 | Peripheral | self-pollination | wet | 0 |
| 535 | Per_4 | Peripheral | self-pollination | wet | 0 |
| 536 | Per_4 | Peripheral | self-pollination | wet | 0 |
| 537 | Per_4 | Peripheral | self-pollination | wet | 0 |
| 538 | Per_4 | Peripheral | self-pollination | wet | 1 |
| 539 | Per_4 | Peripheral | self-pollination | wet | 1 |
| 540 | Per_4 | Peripheral | self-pollination | wet | 1 |
| 541 | Per_5 | Peripheral | between-population cross-pollination | agar | 1 |
| 542 | Per_5 | Peripheral | between-population cross-pollination | agar | 1 |
| 543 | Per_5 | Peripheral | between-population cross-pollination | agar | 0 |
| 544 | Per_5 | Peripheral | between-population cross-pollination | agar | 1 |
| 545 | Per_5 | Peripheral | between-population cross-pollination | agar | 1 |
| 546 | Per_5 | Peripheral | between-population cross-pollination | agar | 1 |
| 547 | Per_5 | Peripheral | between-population cross-pollination | agar | 1 |
| 548 | Per_5 | Peripheral | between-population cross-pollination | agar | 1 |
| 549 | Per_5 | Peripheral | between-population cross-pollination | dry | 1 |
| 550 | Per_5 | Peripheral | between-population cross-pollination | dry | 0 |
| 551 | Per_5 | Peripheral | between-population cross-pollination | dry | 1 |
| 552 | Per_5 | Peripheral | between-population cross-pollination | dry | 0 |
| 553 | Per_5 | Peripheral | between-population cross-pollination | dry | 0 |
| 554 | Per_5 | Peripheral | between-population cross-pollination | dry | 1 |
| 555 | Per_5 | Peripheral | between-population cross-pollination | dry | 1 |
| 556 | Per_5 | Peripheral | between-population cross-pollination | dry | 1 |
| 557 | Per_5 | Peripheral | between-population cross-pollination | wet | 1 |
| 558 | Per_5 | Peripheral | between-population cross-pollination | wet | 1 |
| 559 | Per_5 | Peripheral | between-population cross-pollination | wet | 1 |
| 560 | Per_5 | Peripheral | between-population cross-pollination | wet | 1 |
| 561 | Per_5 | Peripheral | within_population cross-pollination | agar | 1 |
| 562 | Per_5 | Peripheral | within_population cross-pollination | agar | 1 |
| 563 | Per_5 | Peripheral | within_population cross-pollination | agar | 1 |
| 564 | Per_5 | Peripheral | within_population cross-pollination | agar | 1 |
| 565 | Per_5 | Peripheral | within_population cross-pollination | dry | 0 |
| 566 | Per_5 | Peripheral | within_population cross-pollination | dry | 1 |
| 567 | Per_5 | Peripheral | within_population cross-pollination | dry | 0 |
| 568 | Per_5 | Peripheral | within_population cross-pollination | dry | 1 |
| 569 | Per_5 | Peripheral | within_population cross-pollination | dry | 1 |
| 570 | Per_5 | Peripheral | within_population cross-pollination | dry | 1 |
| 571 | Per_5 | Peripheral | within_population cross-pollination | dry | 1 |
| 572 | Per_5 | Peripheral | within_population cross-pollination | dry | 1 |
| 573 | Per_5 | Peripheral | within_population cross-pollination | wet | 0 |
| 574 | Per_5 | Peripheral | within_population cross-pollination | wet | 1 |
| 575 | Per_5 | Peripheral | within_population cross-pollination | wet | 1 |
| 576 | Per_5 | Peripheral | within_population cross-pollination | wet | 1 |
| 577 | Per_5 | Peripheral | within_population cross-pollination | wet | 1 |
| 578 | Per_5 | Peripheral | within_population cross-pollination | wet | 1 |
| 579 | Per_5 | Peripheral | within_population cross-pollination | wet | 1 |
| 580 | Per_5 | Peripheral | within_population cross-pollination | wet | 1 |
| 581 | Per_5 | Peripheral | self-pollination | agar | 0 |
| 582 | Per_5 | Peripheral | self-pollination | agar | 1 |
| 583 | Per_5 | Peripheral | self-pollination | agar | 1 |
| 584 | Per_5 | Peripheral | self-pollination | agar | 1 |
| 585 | Per_5 | Peripheral | self-pollination | agar | 1 |
| 586 | Per_5 | Peripheral | self-pollination | agar | 1 |
| 587 | Per_5 | Peripheral | self-pollination | agar | 1 |
| 588 | Per_5 | Peripheral | self-pollination | agar | 1 |
| 589 | Per_5 | Peripheral | self-pollination | dry | 0 |
| 590 | Per_5 | Peripheral | self-pollination | dry | 0 |
| 591 | Per_5 | Peripheral | self-pollination | dry | 1 |
| 592 | Per_5 | Peripheral | self-pollination | dry | 1 |
| 593 | Per_5 | Peripheral | self-pollination | wet | 0 |
| 594 | Per_5 | Peripheral | self-pollination | wet | 0 |
| 595 | Per_5 | Peripheral | self-pollination | wet | 1 |
| 596 | Per_5 | Peripheral | self-pollination | wet | 0 |
| 597 | Per_5 | Peripheral | self-pollination | wet | 1 |
| 598 | Per_5 | Peripheral | self-pollination | wet | 1 |
| 599 | Per_5 | Peripheral | self-pollination | wet | 1 |
| 600 | Per_5 | Peripheral | self-pollination | wet | 1 |

**Table S3.2. Radicle length and shoot size after 21 observation days.**

| Id_Plant | Family | Population_type | Pollination_treatment | Stress_treatment | ROOT[mm] | SHOOT[mm] |
| --- | --- | --- | --- | --- | --- | --- |
| 1 | Cor_1 | core | between-population cross-pollination | dry | 15.17 | 24.5 |
| 2 | Cor_1 | core | between-population cross-pollination | dry | 7.27 | 28.6 |
| 3 | Cor_1 | core | between-population cross-pollination | dry | 21.2 | 36.73 |
| 4 | Cor_1 | core | between-population cross-pollination | dry | 28.76 | 45.32 |
| 5 | Cor_1 | core | between-population cross-pollination | dry | 30.26 | 61.28 |
| 6 | Cor_1 | core | between-population cross-pollination | dry | 24.75 | 37.06 |
| 7 | Cor_1 | core | between-population cross-pollination | dry | 13.55 | 19.3 |
| 8 | Cor_1 | core | between-population cross-pollination | wet | 11.52 | 25.22 |
| 9 | Cor_1 | core | between-population cross-pollination | wet | 22.17 | 38.65 |
| 10 | Cor_1 | core | between-population cross-pollination | wet | 12.9 | 30.8 |
| 11 | Cor_1 | core | between-population cross-pollination | wet | 15.65 | 30.57 |
| 12 | Cor_1 | core | between-population cross-pollination | wet | 18.57 | 32.93 |
| 13 | Cor_1 | core | within-population cross-pollination | dry | 15.58 | 35.81 |
| 14 | Cor_1 | core | within-population cross-pollination | dry | 5.55 | 17.31 |
| 15 | Cor_1 | core | within-population cross-pollination | dry | 15.86 | 29.05 |
| 16 | Cor_1 | core | within-population cross-pollination | dry | 22.63 | 35.57 |
| 17 | Cor_1 | core | within-population cross-pollination | dry | 14.67 | 33.37 |
| 18 | Cor_1 | core | within-population cross-pollination | dry | 21.71 | 33.94 |
| 19 | Cor_1 | core | within-population cross-pollination | wet | 20.39 | 33.4 |
| 20 | Cor_1 | core | within-population cross-pollination | wet | 22.81 | 38 |
| 21 | Cor_1 | core | within-population cross-pollination | wet | 21.44 | 36.73 |
| 22 | Cor_1 | core | within-population cross-pollination | wet | 24.5 | 38.51 |
| 23 | Cor_1 | core | within-population cross-pollination | wet | 26.8 | 45.88 |
| 24 | Cor_1 | core | within-population cross-pollination | wet | 17.08 | 36.06 |
| 25 | Cor_1 | core | self-pollination | dry | 11.64 | 28.22 |
| 26 | Cor_1 | core | self-pollination | dry | 9.49 | 24.67 |
| 27 | Cor_1 | core | self-pollination | dry | 17.32 | 29.08 |
| 28 | Cor_1 | core | self-pollination | dry | 23.66 | 43.2 |
| 29 | Cor_1 | core | self-pollination | wet | 15.2 | 32.96 |
| 30 | Cor_1 | core | self-pollination | wet | 15.59 | 23.16 |
| 31 | Cor_1 | core | self-pollination | wet | 9.21 | 18.29 |
| 32 | Cor_1 | core | self-pollination | wet | 18.81 | 34.27 |
| 33 | Cor_1 | core | self-pollination | wet | 19.85 | 31.11 |
| 34 | Cor_2 | core | between-population cross-pollination | dry | 15.92 | 30.08 |
| 35 | Cor_2 | core | between-population cross-pollination | dry | 11.27 | 26.77 |
| 36 | Cor_2 | core | between-population cross-pollination | dry | 9.74 | 22.54 |
| 37 | Cor_2 | core | between-population cross-pollination | dry | 15.59 | 39.11 |
| 38 | Cor_2 | core | between-population cross-pollination | dry | 10.29 | 39.38 |
| 39 | Cor_2 | core | between-population cross-pollination | dry | 34.96 | 62.47 |
| 40 | Cor_2 | core | between-population cross-pollination | dry | 27.91 | 52.32 |
| 41 | Cor_2 | core | between-population cross-pollination | wet | 10.66 | 24.12 |
| 42 | Cor_2 | core | between-population cross-pollination | wet | 9.6 | 25.58 |
| 43 | Cor_2 | core | between-population cross-pollination | wet | 23.25 | 38.64 |
| 44 | Cor_2 | core | between-population cross-pollination | wet | 18.81 | 32.81 |
| 45 | Cor_2 | core | between-population cross-pollination | wet | 13.65 | 24.71 |
| 46 | Cor_2 | core | within-population cross-pollination | dry | 16.85 | 32.96 |
| 47 | Cor_2 | core | within-population cross-pollination | dry | 17.81 | 35.39 |
| 48 | Cor_2 | core | within-population cross-pollination | dry | 13.7 | 33.4 |
| 49 | Cor_2 | core | within-population cross-pollination | dry | 16.02 | 29.61 |
| 50 | Cor_2 | core | within-population cross-pollination | dry | 23.03 | 37.06 |
| 51 | Cor_2 | core | within-population cross-pollination | dry | 19.16 | 33.07 |
| 52 | Cor_2 | core | within-population cross-pollination | wet | 42.05 | 52.41 |
| 53 | Cor_2 | core | within-population cross-pollination | wet | 23.43 | 35.27 |
| 54 | Cor_2 | core | within-population cross-pollination | wet | 14.15 | 19.9 |
| 55 | Cor_2 | core | within-population cross-pollination | wet | 22.96 | 31.28 |
| 56 | Cor_2 | core | within-population cross-pollination | wet | 32.01 | 51.14 |
| 57 | Cor_2 | core | within-population cross-pollination | wet | 30.23 | 52.61 |
| 58 | Cor_2 | core | self-pollination | dry | 26.11 | 36.26 |
| 59 | Cor_2 | core | self-pollination | dry | 12.5 | 31.12 |
| 60 | Cor_2 | core | self-pollination | dry | 23.07 | 37.84 |
| 61 | Cor_2 | core | self-pollination | dry | 22.63 | 35.91 |
| 62 | Cor_2 | core | self-pollination | wet | 7 | 17.22 |
| 63 | Cor_2 | core | self-pollination | wet | 17.94 | 32.35 |
| 64 | Cor_2 | core | self-pollination | wet | 16.13 | 27.22 |
| 65 | Cor_2 | core | self-pollination | wet | 25.23 | 34.94 |
| 66 | Cor_3 | core | between-population cross-pollination | dry | 13.35 | 27.36 |
| 67 | Cor_3 | core | between-population cross-pollination | dry | 14.55 | 32.48 |
| 68 | Cor_3 | core | between-population cross-pollination | dry | 11.94 | 27.07 |
| 69 | Cor_3 | core | between-population cross-pollination | dry | 17.51 | 40.59 |
| 70 | Cor_3 | core | between-population cross-pollination | dry | 39 | 60.34 |
| 71 | Cor_3 | core | between-population cross-pollination | dry | 38.96 | 61.75 |
| 72 | Cor_3 | core | between-population cross-pollination | dry | 15.12 | 40.34 |
| 73 | Cor_3 | core | between-population cross-pollination | wet | 19.3 | 41.03 |
| 74 | Cor_3 | core | between-population cross-pollination | wet | 17.15 | 27.79 |
| 75 | Cor_3 | core | between-population cross-pollination | wet | 18.86 | 36.25 |
| 76 | Cor_3 | core | between-population cross-pollination | wet | 13.93 | 29.92 |
| 77 | Cor_3 | core | between-population cross-pollination | wet | 23.84 | 40.13 |
| 78 | Cor_3 | core | within-population cross-pollination | dry | 10.96 | 30.13 |
| 79 | Cor_3 | core | within-population cross-pollination | dry | 15.69 | 31.63 |
| 80 | Cor_3 | core | within-population cross-pollination | dry | 20.25 | 30.54 |
| 81 | Cor_3 | core | within-population cross-pollination | dry | 23.99 | 43.78 |
| 82 | Cor_3 | core | within-population cross-pollination | dry | 10.33 | 28.18 |
| 83 | Cor_3 | core | within-population cross-pollination | dry | 23.81 | 35.44 |
| 84 | Cor_3 | core | within-population cross-pollination | wet | 32.18 | 48.34 |
| 85 | Cor_3 | core | within-population cross-pollination | wet | 31.12 | 45.81 |
| 86 | Cor_3 | core | within-population cross-pollination | wet | 16.61 | 30.87 |
| 87 | Cor_3 | core | within-population cross-pollination | wet | 27.6 | 44.75 |
| 88 | Cor_3 | core | within-population cross-pollination | wet | 22.65 | 44.26 |
| 89 | Cor_3 | core | within-population cross-pollination | wet | 13.69 | 25.61 |
| 90 | Cor_3 | core | within-population cross-pollination | wet | 18.78 | 38.74 |
| 91 | Cor_3 | core | self-pollination | dry | 7.88 | 17.89 |
| 92 | Cor_3 | core | self-pollination | dry | 5.11 | 11.03 |
| 93 | Cor_3 | core | self-pollination | dry | 18.11 | 34.66 |
| 94 | Cor_3 | core | self-pollination | dry | 3.41 | 7.42 |
| 95 | Cor_3 | core | self-pollination | wet | 24.8 | 37.53 |
| 96 | Cor_3 | core | self-pollination | wet | 10.52 | 17.68 |
| 97 | Cor_3 | core | self-pollination | wet | 29.52 | 41.37 |
| 98 | Cor_3 | core | self-pollination | wet | 17.34 | 35.56 |
| 99 | Cor_4 | core | between-population cross-pollination | dry | 21.91 | 32.16 |
| 100 | Cor_4 | core | between-population cross-pollination | dry | 9.07 | 23.5 |
| 101 | Cor_4 | core | between-population cross-pollination | dry | 8.63 | 22.48 |
| 102 | Cor_4 | core | between-population cross-pollination | dry | 18.35 | 31.21 |
| 103 | Cor_4 | core | between-population cross-pollination | dry | 32.43 | 60.64 |
| 104 | Cor_4 | core | between-population cross-pollination | dry | 21.13 | 43.96 |
| 105 | Cor_4 | core | between-population cross-pollination | wet | 14.47 | 30.72 |
| 106 | Cor_4 | core | between-population cross-pollination | wet | 24.14 | 37.14 |
| 107 | Cor_4 | core | between-population cross-pollination | wet | 18.47 | 32.93 |
| 108 | Cor_4 | core | between-population cross-pollination | wet | 18.73 | 33.84 |
| 109 | Cor_4 | core | between-population cross-pollination | wet | 13.65 | 29.49 |
| 110 | Cor_4 | core | between-population cross-pollination | wet | 27.44 | 43.5 |
| 111 | Cor_4 | core | within-population cross-pollination | dry | 24.65 | 44.19 |
| 112 | Cor_4 | core | within-population cross-pollination | dry | 8.91 | 21.77 |
| 113 | Cor_4 | core | within-population cross-pollination | dry | 22.51 | 37.59 |
| 114 | Cor_4 | core | within-population cross-pollination | dry | 19.35 | 29.78 |
| 115 | Cor_4 | core | within-population cross-pollination | dry | 19.95 | 37.62 |
| 116 | Cor_4 | core | within-population cross-pollination | dry | 30.9 | 52.3 |
| 117 | Cor_4 | core | within-population cross-pollination | wet | 14.11 | 30.78 |
| 118 | Cor_4 | core | within-population cross-pollination | wet | 26.3 | 42.97 |
| 119 | Cor_4 | core | within-population cross-pollination | wet | 16.6 | 23.38 |
| 120 | Cor_4 | core | within-population cross-pollination | wet | 33.42 | 48.11 |
| 121 | Cor_4 | core | within-population cross-pollination | wet | 27.69 | 43.2 |
| 122 | Cor_4 | core | within-population cross-pollination | wet | 19.99 | 35.18 |
| 123 | Cor_4 | core | self-pollination | dry | 18.11 | 32.67 |
| 124 | Cor_4 | core | self-pollination | dry | 19.53 | 38.07 |
| 125 | Cor_4 | core | self-pollination | dry | 22.92 | 39.99 |
| 126 | Cor_4 | core | self-pollination | dry | 9.62 | 23.14 |
| 127 | Cor_4 | core | self-pollination | wet | 6.73 | 17.26 |
| 128 | Cor_4 | core | self-pollination | wet | 17.42 | 34.63 |
| 129 | Cor_4 | core | self-pollination | wet | 16.9 | 39.28 |
| 130 | Cor_4 | core | self-pollination | wet | 12.5 | 29.67 |
| 131 | Cor_5 | core | between-population cross-pollination | dry | 15 | 29.08 |
| 132 | Cor_5 | core | between-population cross-pollination | dry | 20.9 | 34.05 |
| 133 | Cor_5 | core | between-population cross-pollination | dry | 12.63 | 30.13 |
| 134 | Cor_5 | core | between-population cross-pollination | dry | 17.04 | 45.23 |
| 135 | Cor_5 | core | between-population cross-pollination | dry | 21.32 | 48.11 |
| 136 | Cor_5 | core | between-population cross-pollination | dry | 16.53 | 39.44 |
| 137 | Cor_5 | core | between-population cross-pollination | wet | 21.33 | 45.71 |
| 138 | Cor_5 | core | between-population cross-pollination | wet | 18.55 | 38.64 |
| 139 | Cor_5 | core | between-population cross-pollination | wet | 18.98 | 37.25 |
| 140 | Cor_5 | core | between-population cross-pollination | wet | 18.48 | 35.72 |
| 141 | Cor_5 | core | between-population cross-pollination | wet | 14.43 | 31.94 |
| 142 | Cor_5 | core | between-population cross-pollination | wet | 38.6 | 61.83 |
| 143 | Cor_5 | core | within-population cross-pollination | dry | 20.82 | 42.01 |
| 144 | Cor_5 | core | within-population cross-pollination | dry | 19.37 | 41.29 |
| 145 | Cor_5 | core | within-population cross-pollination | dry | 19.21 | 42.61 |
| 146 | Cor_5 | core | within-population cross-pollination | dry | 15.97 | 28.54 |
| 147 | Cor_5 | core | within-population cross-pollination | dry | 7.48 | 21.45 |
| 148 | Cor_5 | core | within-population cross-pollination | dry | 28.16 | 43.02 |
| 149 | Cor_5 | core | within-population cross-pollination | wet | 29.99 | 41.8 |
| 150 | Cor_5 | core | within-population cross-pollination | wet | 23.32 | 40.95 |
| 151 | Cor_5 | core | within-population cross-pollination | wet | 16.32 | 28.92 |
| 152 | Cor_5 | core | within-population cross-pollination | wet | 29.6 | 49.13 |
| 153 | Cor_5 | core | within-population cross-pollination | wet | 20.71 | 36.38 |
| 154 | Cor_5 | core | within-population cross-pollination | wet | 23.61 | 42.73 |
| 155 | Cor_5 | core | self-pollination | dry | 15.97 | 36.36 |
| 156 | Cor_5 | core | self-pollination | dry | 12.78 | 32.12 |
| 157 | Cor_5 | core | self-pollination | dry | 9.37 | 30.39 |
| 158 | Cor_5 | core | self-pollination | dry | 4.32 | 11.45 |
| 159 | Cor_5 | core | self-pollination | wet | 11.49 | 32.19 |
| 160 | Cor_5 | core | self-pollination | wet | 10.52 | 18.41 |
| 161 | Cor_5 | core | self-pollination | wet | 12.44 | 26.58 |
| 162 | Cor_5 | core | self-pollination | wet | 11.34 | 21.81 |
| 163 | Per_1 | Peripheral | between-population cross-pollination | dry | 17.97 | 32.55 |
| 164 | Per_1 | Peripheral | between-population cross-pollination | dry | 25.04 | 45.55 |
| 165 | Per_1 | Peripheral | between-population cross-pollination | dry | 20.51 | 36.58 |
| 166 | Per_1 | Peripheral | between-population cross-pollination | dry | 17.71 | 32.93 |
| 167 | Per_1 | Peripheral | between-population cross-pollination | dry | 26.94 | 42.24 |
| 168 | Per_1 | Peripheral | between-population cross-pollination | wet | 15.47 | 43.36 |
| 169 | Per_1 | Peripheral | between-population cross-pollination | wet | 13.36 | 49.11 |
| 170 | Per_1 | Peripheral | between-population cross-pollination | wet | 22.33 | 38.65 |
| 171 | Per_1 | Peripheral | between-population cross-pollination | wet | 24.22 | 34.06 |
| 172 | Per_1 | Peripheral | between-population cross-pollination | wet | 12.8 | 34.58 |
| 173 | Per_1 | Peripheral | between-population cross-pollination | wet | 28.43 | 34.74 |
| 174 | Per_1 | Peripheral | within-population cross-pollination | dry | 20.9 | 39.16 |
| 175 | Per_1 | Peripheral | within-population cross-pollination | dry | 23.18 | 39.99 |
| 176 | Per_1 | Peripheral | within-population cross-pollination | dry | 24.77 | 45.08 |
| 177 | Per_1 | Peripheral | within-population cross-pollination | dry | 23.27 | 36.45 |
| 178 | Per_1 | Peripheral | within-population cross-pollination | wet | 23.23 | 37.17 |
| 179 | Per_1 | Peripheral | within-population cross-pollination | wet | 20.65 | 26.11 |
| 180 | Per_1 | Peripheral | within-population cross-pollination | wet | 23.79 | 40.07 |
| 181 | Per_1 | Peripheral | within-population cross-pollination | wet | 19.87 | 44.78 |
| 182 | Per_1 | Peripheral | within-population cross-pollination | wet | 16.22 | 25.04 |
| 183 | Per_1 | Peripheral | within-population cross-pollination | wet | 22.5 | 49.31 |
| 184 | Per_1 | Peripheral | within-population cross-pollination | wet | 12.22 | na |
| 185 | Per_1 | Peripheral | self-pollination | dry | 2.08 | 3.6 |
| 186 | Per_1 | Peripheral | self-pollination | dry | 24.95 | 46.28 |
| 187 | Per_1 | Peripheral | self-pollination | dry | 22.36 | 39.13 |
| 188 | Per_1 | Peripheral | self-pollination | dry | 23.72 | 43.29 |
| 189 | Per_1 | Peripheral | self-pollination | wet | 27.83 | 41.67 |
| 190 | Per_1 | Peripheral | self-pollination | wet | 1.99 | 6.95 |
| 191 | Per_1 | Peripheral | self-pollination | wet | 18.18 | 34.57 |
| 192 | Per_1 | Peripheral | self-pollination | wet | 16.28 | 24.86 |
| 193 | Per_1 | Peripheral | self-pollination | wet | 16.18 | 40.14 |
| 194 | Per_2 | Peripheral | between-population cross-pollination | dry | 10.48 | 29.03 |
| 195 | Per_2 | Peripheral | between-population cross-pollination | dry | 17.74 | 25.38 |
| 196 | Per_2 | Peripheral | between-population cross-pollination | dry | 10.39 | 21.08 |
| 197 | Per_2 | Peripheral | between-population cross-pollination | dry | 18.79 | 33.96 |
| 198 | Per_2 | Peripheral | between-population cross-pollination | dry | 17.5 | 32.58 |
| 199 | Per_2 | Peripheral | between-population cross-pollination | wet | 23.11 | 50.68 |
| 200 | Per_2 | Peripheral | between-population cross-pollination | wet | 15.84 | 18.62 |
| 201 | Per_2 | Peripheral | between-population cross-pollination | wet | 11.33 | 34.57 |
| 202 | Per_2 | Peripheral | between-population cross-pollination | wet | 25.36 | 39.24 |
| 203 | Per_2 | Peripheral | between-population cross-pollination | wet | 39.14 | 44.58 |
| 204 | Per_2 | Peripheral | within-population cross-pollination | dry | 12.85 | 35.94 |
| 205 | Per_2 | Peripheral | within-population cross-pollination | dry | 9.43 | 23.94 |
| 206 | Per_2 | Peripheral | within-population cross-pollination | dry | 20.87 | 34.08 |
| 207 | Per_2 | Peripheral | within-population cross-pollination | wet | 28.19 | 41.31 |
| 208 | Per_2 | Peripheral | within-population cross-pollination | wet | 8.18 | 29.92 |
| 209 | Per_2 | Peripheral | within-population cross-pollination | wet | 12.04 | 28.04 |
| 210 | Per_2 | Peripheral | within-population cross-pollination | wet | 23.42 | 42.42 |
| 211 | Per_2 | Peripheral | within-population cross-pollination | wet | 24.33 | 49.02 |
| 212 | Per_2 | Peripheral | within-population cross-pollination | wet | 25.35 | 38.92 |
| 213 | Per_2 | Peripheral | within-population cross-pollination | wet | 23.49 | na |
| 214 | Per_2 | Peripheral | self-pollination | dry | 11.6 | 25.14 |
| 215 | Per_2 | Peripheral | self-pollination | dry | 11.25 | 27.84 |
| 216 | Per_2 | Peripheral | self-pollination | dry | 17.22 | 34.76 |
| 217 | Per_2 | Peripheral | self-pollination | dry | 17.82 | 33.92 |
| 218 | Per_2 | Peripheral | self-pollination | wet | 26.55 | 37.68 |
| 219 | Per_2 | Peripheral | self-pollination | wet | 14.69 | 30.8 |
| 220 | Per_2 | Peripheral | self-pollination | wet | 8.86 | 19.63 |
| 221 | Per_2 | Peripheral | self-pollination | wet | 17.86 | 26.48 |
| 222 | Per_2 | Peripheral | self-pollination | wet | 17.19 | 34.7 |
| 223 | Per_3 | Peripheral | between-population cross-pollination | dry | 10.75 | 28.59 |
| 224 | Per_3 | Peripheral | between-population cross-pollination | dry | 22.76 | 37.13 |
| 225 | Per_3 | Peripheral | between-population cross-pollination | dry | 12.03 | 29.64 |
| 226 | Per_3 | Peripheral | between-population cross-pollination | dry | 12.84 | 23.87 |
| 227 | Per_3 | Peripheral | between-population cross-pollination | dry | 18.73 | 31.57 |
| 228 | Per_3 | Peripheral | between-population cross-pollination | wet | 13.89 | 55.4 |
| 229 | Per_3 | Peripheral | between-population cross-pollination | wet | 19.06 | 33.18 |
| 230 | Per_3 | Peripheral | between-population cross-pollination | wet | 34.28 | 41.93 |
| 231 | Per_3 | Peripheral | between-population cross-pollination | wet | 16.87 | 52.13 |
| 232 | Per_3 | Peripheral | between-population cross-pollination | wet | 21.57 | 40.85 |
| 233 | Per_3 | Peripheral | within-population cross-pollination | dry | 19.49 | 39.92 |
| 234 | Per_3 | Peripheral | within-population cross-pollination | dry | 23.39 | 42.3 |
| 235 | Per_3 | Peripheral | within-population cross-pollination | dry | 24.72 | 40.78 |
| 236 | Per_3 | Peripheral | within-population cross-pollination | wet | 30.73 | 31.43 |
| 237 | Per_3 | Peripheral | within-population cross-pollination | wet | 22.48 | 33.02 |
| 238 | Per_3 | Peripheral | within-population cross-pollination | wet | 24.65 | 48.22 |
| 239 | Per_3 | Peripheral | within-population cross-pollination | wet | 29.2 | 29.61 |
| 240 | Per_3 | Peripheral | within-population cross-pollination | wet | 23.21 | 41.29 |
| 241 | Per_3 | Peripheral | within-population cross-pollination | wet | 17.48 | na |
| 242 | Per_3 | Peripheral | self-pollination | dry | 16.05 | 31.15 |
| 243 | Per_3 | Peripheral | self-pollination | dry | 11.68 | 31.44 |
| 244 | Per_3 | Peripheral | self-pollination | dry | 12.28 | 27.96 |
| 245 | Per_3 | Peripheral | self-pollination | dry | 12.53 | 25.02 |
| 246 | Per_3 | Peripheral | self-pollination | wet | 17.33 | 33.44 |
| 247 | Per_3 | Peripheral | self-pollination | wet | 16.72 | 27.82 |
| 248 | Per_3 | Peripheral | self-pollination | wet | 17.94 | 42 |
| 249 | Per_3 | Peripheral | self-pollination | wet | 5.59 | 14.99 |
| 250 | Per_4 | Peripheral | between-population cross-pollination | dry | 0 | 7.88 |
| 251 | Per_4 | Peripheral | between-population cross-pollination | dry | 25.17 | 45.96 |
| 252 | Per_4 | Peripheral | between-population cross-pollination | dry | 26.79 | 43.15 |
| 253 | Per_4 | Peripheral | between-population cross-pollination | dry | 19.62 | 34.7 |
| 254 | Per_4 | Peripheral | between-population cross-pollination | dry | 17.6 | 32.09 |
| 255 | Per_4 | Peripheral | between-population cross-pollination | wet | 19.32 | 34.18 |
| 256 | Per_4 | Peripheral | between-population cross-pollination | wet | 28.12 | 42.17 |
| 257 | Per_4 | Peripheral | between-population cross-pollination | wet | 20.52 | 46.22 |
| 258 | Per_4 | Peripheral | between-population cross-pollination | wet | 14.68 | 36.99 |
| 259 | Per_4 | Peripheral | between-population cross-pollination | wet | 19.64 | 20.5 |
| 260 | Per_4 | Peripheral | within-population cross-pollination | dry | 18.14 | 30.88 |
| 261 | Per_4 | Peripheral | within-population cross-pollination | dry | 25.71 | 46.48 |
| 262 | Per_4 | Peripheral | within-population cross-pollination | dry | 22.73 | 43.4 |
| 263 | Per_4 | Peripheral | within-population cross-pollination | wet | 19.94 | 35.53 |
| 264 | Per_4 | Peripheral | within-population cross-pollination | wet | 22.47 | 38.63 |
| 265 | Per_4 | Peripheral | within-population cross-pollination | wet | 24.06 | 34.33 |
| 266 | Per_4 | Peripheral | within-population cross-pollination | wet | 17.03 | 30.89 |
| 267 | Per_4 | Peripheral | within-population cross-pollination | wet | 7.75 | 38.04 |
| 268 | Per_4 | Peripheral | within-population cross-pollination | wet | 20.56 | na |
| 269 | Per_4 | Peripheral | self-pollination | dry | 14.28 | 29.49 |
| 270 | Per_4 | Peripheral | self-pollination | dry | 14.2 | 27.64 |
| 271 | Per_4 | Peripheral | self-pollination | dry | 12.04 | 26 |
| 272 | Per_4 | Peripheral | self-pollination | wet | 17.5 | 27.59 |
| 273 | Per_4 | Peripheral | self-pollination | wet | 17.62 | 29.52 |
| 274 | Per_4 | Peripheral | self-pollination | wet | 15.36 | 36.11 |
| 275 | Per_4 | Peripheral | self-pollination | wet | 5.47 | 13.9 |
| 276 | Per_5 | Peripheral | between-population cross-pollination | dry | 23.98 | 39.07 |
| 277 | Per_5 | Peripheral | between-population cross-pollination | dry | 19.77 | 37.53 |
| 278 | Per_5 | Peripheral | between-population cross-pollination | dry | 20.93 | 43.02 |
| 279 | Per_5 | Peripheral | between-population cross-pollination | dry | 22.36 | 35.67 |
| 280 | Per_5 | Peripheral | between-population cross-pollination | dry | 22.09 | 35.46 |
| 281 | Per_5 | Peripheral | between-population cross-pollination | wet | 23.34 | 39.47 |
| 282 | Per_5 | Peripheral | between-population cross-pollination | wet | 12.51 | 36.68 |
| 283 | Per_5 | Peripheral | between-population cross-pollination | wet | 34.1 | 47.37 |
| 284 | Per_5 | Peripheral | between-population cross-pollination | wet | 20.71 | 41.85 |
| 285 | Per_5 | Peripheral | between-population cross-pollination | wet | 19.31 | 38.26 |
| 286 | Per_5 | Peripheral | between-population cross-pollination | wet | 28.72 | 41.6 |
| 287 | Per_5 | Peripheral | within-population cross-pollination | dry | 10.4 | 32.85 |
| 288 | Per_5 | Peripheral | within-population cross-pollination | dry | 19.64 | 38.99 |
| 289 | Per_5 | Peripheral | within-population cross-pollination | dry | 17.87 | 28.35 |
| 290 | Per_5 | Peripheral | within-population cross-pollination | dry | 12.48 | 31.7 |
| 291 | Per_5 | Peripheral | within-population cross-pollination | wet | 18.31 | 37.33 |
| 292 | Per_5 | Peripheral | within-population cross-pollination | wet | 20.64 | 32.47 |
| 293 | Per_5 | Peripheral | within-population cross-pollination | wet | 24.84 | 50.76 |
| 294 | Per_5 | Peripheral | within-population cross-pollination | wet | 24.77 | 33.88 |
| 295 | Per_5 | Peripheral | within-population cross-pollination | wet | 19.58 | 36.66 |
| 296 | Per_5 | Peripheral | within-population cross-pollination | wet | 20.7 | na |
| 297 | Per_5 | Peripheral | within-population cross-pollination | wet | 20.9 | na |
| 298 | Per_5 | Peripheral | self-pollination | dry | 12.32 | 21.21 |
| 299 | Per_5 | Peripheral | self-pollination | dry | 12.24 | 23.02 |
| 300 | Per_5 | Peripheral | self-pollination | dry | 14.25 | 28.3 |
| 301 | Per_5 | Peripheral | self-pollination | dry | 21.51 | na |
| 302 | Per_5 | Peripheral | self-pollination | wet | 16.15 | 34.11 |
| 303 | Per_5 | Peripheral | self-pollination | wet | 21.34 | 38.61 |
| 304 | Per_5 | Peripheral | self-pollination | wet | 21.86 | 34.82 |
| 305 | Per_5 | Peripheral | self-pollination | wet | 19.28 | 35.75 |
| 306 | Per_5 | Peripheral | self-pollination | wet | 18.85 | 31 |

**Table S3.3. Survival data for 21-days old seedlings.**

| Id_plant | Family | Population_type | Pollination_treatment | Stress_treatment | SURV[1,0] |
| --- | --- | --- | --- | --- | --- |
| 1 | Cor_1 | core | between-population cross-pollination | dry | 1 |
| 2 | Cor_1 | core | between-population cross-pollination | dry | 1 |
| 3 | Cor_1 | core | between-population cross-pollination | dry | 1 |
| 4 | Cor_1 | core | between-population cross-pollination | dry | 1 |
| 5 | Cor_1 | core | between-population cross-pollination | dry | 1 |
| 6 | Cor_1 | core | between-population cross-pollination | dry | 0 |
| 7 | Cor_1 | core | between-population cross-pollination | dry | 0 |
| 8 | Cor_1 | core | between-population cross-pollination | dry | 1 |
| 9 | Cor_1 | core | between-population cross-pollination | wet | 0 |
| 10 | Cor_1 | core | between-population cross-pollination | wet | 1 |
| 11 | Cor_1 | core | between-population cross-pollination | wet | 1 |
| 12 | Cor_1 | core | between-population cross-pollination | wet | 0 |
| 13 | Cor_1 | core | between-population cross-pollination | wet | 1 |
| 14 | Cor_1 | core | between-population cross-pollination | wet | 1 |
| 15 | Cor_1 | core | between-population cross-pollination | wet | 1 |
| 16 | Cor_1 | core | between-population cross-pollination | wet | 1 |
| 17 | Cor_1 | core | within-population cross-pollination | dry | 1 |
| 18 | Cor_1 | core | within-population cross-pollination | dry | 1 |
| 19 | Cor_1 | core | within-population cross-pollination | dry | 1 |
| 20 | Cor_1 | core | within-population cross-pollination | dry | 1 |
| 21 | Cor_1 | core | within-population cross-pollination | dry | 1 |
| 22 | Cor_1 | core | within-population cross-pollination | dry | 0 |
| 23 | Cor_1 | core | within-population cross-pollination | dry | 1 |
| 24 | Cor_1 | core | within-population cross-pollination | dry | 0 |
| 25 | Cor_1 | core | within-population cross-pollination | dry | 0 |
| 26 | Cor_1 | core | within-population cross-pollination | wet | 1 |
| 27 | Cor_1 | core | within-population cross-pollination | wet | 1 |
| 28 | Cor_1 | core | within-population cross-pollination | wet | 1 |
| 29 | Cor_1 | core | within-population cross-pollination | wet | 1 |
| 30 | Cor_1 | core | within-population cross-pollination | wet | 1 |
| 31 | Cor_1 | core | within-population cross-pollination | wet | 1 |
| 32 | Cor_1 | core | within-population cross-pollination | wet | 1 |
| 33 | Cor_1 | core | within-population cross-pollination | wet | 1 |
| 34 | Cor_1 | core | self-pollination | dry | 1 |
| 35 | Cor_1 | core | self-pollination | dry | 0 |
| 36 | Cor_1 | core | self-pollination | dry | 1 |
| 37 | Cor_1 | core | self-pollination | dry | 0 |
| 38 | Cor_1 | core | self-pollination | dry | 1 |
| 39 | Cor_1 | core | self-pollination | dry | 0 |
| 40 | Cor_1 | core | self-pollination | dry | 0 |
| 41 | Cor_1 | core | self-pollination | wet | 0 |
| 42 | Cor_1 | core | self-pollination | wet | 0 |
| 43 | Cor_1 | core | self-pollination | wet | 1 |
| 44 | Cor_1 | core | self-pollination | wet | 1 |
| 45 | Cor_1 | core | self-pollination | wet | 0 |
| 46 | Cor_1 | core | self-pollination | wet | 1 |
| 47 | Cor_1 | core | self-pollination | wet | 1 |
| 48 | Cor_1 | core | self-pollination | wet | 1 |
| 49 | Cor_1 | core | self-pollination | wet | 0 |
| 50 | Cor_2 | core | between-population cross-pollination | dry | 1 |
| 51 | Cor_2 | core | between-population cross-pollination | dry | 1 |
| 52 | Cor_2 | core | between-population cross-pollination | dry | 1 |
| 53 | Cor_2 | core | between-population cross-pollination | dry | 0 |
| 54 | Cor_2 | core | between-population cross-pollination | dry | 1 |
| 55 | Cor_2 | core | between-population cross-pollination | dry | 1 |
| 56 | Cor_2 | core | between-population cross-pollination | dry | 1 |
| 57 | Cor_2 | core | between-population cross-pollination | dry | 1 |
| 58 | Cor_2 | core | between-population cross-pollination | wet | 1 |
| 59 | Cor_2 | core | between-population cross-pollination | wet | 1 |
| 60 | Cor_2 | core | between-population cross-pollination | wet | 1 |
| 61 | Cor_2 | core | between-population cross-pollination | wet | 0 |
| 62 | Cor_2 | core | between-population cross-pollination | wet | 1 |
| 63 | Cor_2 | core | between-population cross-pollination | wet | 1 |
| 64 | Cor_2 | core | between-population cross-pollination | wet | 1 |
| 65 | Cor_2 | core | between-population cross-pollination | wet | 1 |
| 66 | Cor_2 | core | within-population cross-pollination | dry | 1 |
| 67 | Cor_2 | core | within-population cross-pollination | dry | 0 |
| 68 | Cor_2 | core | within-population cross-pollination | dry | 1 |
| 69 | Cor_2 | core | within-population cross-pollination | dry | 0 |
| 70 | Cor_2 | core | within-population cross-pollination | dry | 1 |
| 71 | Cor_2 | core | within-population cross-pollination | dry | 1 |
| 72 | Cor_2 | core | within-population cross-pollination | dry | 0 |
| 73 | Cor_2 | core | within-population cross-pollination | dry | 1 |
| 74 | Cor_2 | core | within-population cross-pollination | wet | 1 |
| 75 | Cor_2 | core | within-population cross-pollination | wet | 1 |
| 76 | Cor_2 | core | within-population cross-pollination | wet | 1 |
| 77 | Cor_2 | core | within-population cross-pollination | wet | 1 |
| 78 | Cor_2 | core | within-population cross-pollination | wet | 1 |
| 79 | Cor_2 | core | within-population cross-pollination | wet | 1 |
| 80 | Cor_2 | core | within-population cross-pollination | wet | 1 |
| 81 | Cor_2 | core | within-population cross-pollination | wet | 1 |
| 82 | Cor_2 | core | self-pollination | dry | 1 |
| 83 | Cor_2 | core | self-pollination | dry | 1 |
| 84 | Cor_2 | core | self-pollination | dry | 1 |
| 85 | Cor_2 | core | self-pollination | dry | 0 |
| 86 | Cor_2 | core | self-pollination | dry | 1 |
| 87 | Cor_2 | core | self-pollination | dry | 1 |
| 88 | Cor_2 | core | self-pollination | dry | 0 |
| 89 | Cor_2 | core | self-pollination | dry | 0 |
| 90 | Cor_2 | core | self-pollination | wet | 0 |
| 91 | Cor_2 | core | self-pollination | wet | 1 |
| 92 | Cor_2 | core | self-pollination | wet | 0 |
| 93 | Cor_2 | core | self-pollination | wet | 1 |
| 94 | Cor_2 | core | self-pollination | wet | 1 |
| 95 | Cor_2 | core | self-pollination | wet | 1 |
| 96 | Cor_2 | core | self-pollination | wet | 0 |
| 97 | Cor_2 | core | self-pollination | wet | 1 |
| 98 | Cor_3 | core | between-population cross-pollination | dry | 1 |
| 99 | Cor_3 | core | between-population cross-pollination | dry | 0 |
| 100 | Cor_3 | core | between-population cross-pollination | dry | 1 |
| 101 | Cor_3 | core | between-population cross-pollination | dry | 1 |
| 102 | Cor_3 | core | between-population cross-pollination | dry | 0 |
| 103 | Cor_3 | core | between-population cross-pollination | dry | 1 |
| 104 | Cor_3 | core | between-population cross-pollination | dry | 1 |
| 105 | Cor_3 | core | between-population cross-pollination | dry | 1 |
| 106 | Cor_3 | core | between-population cross-pollination | wet | 1 |
| 107 | Cor_3 | core | between-population cross-pollination | wet | 1 |
| 108 | Cor_3 | core | between-population cross-pollination | wet | 1 |
| 109 | Cor_3 | core | between-population cross-pollination | wet | 0 |
| 110 | Cor_3 | core | between-population cross-pollination | wet | 1 |
| 111 | Cor_3 | core | between-population cross-pollination | wet | 1 |
| 112 | Cor_3 | core | between-population cross-pollination | wet | 1 |
| 113 | Cor_3 | core | between-population cross-pollination | wet | 0 |
| 114 | Cor_3 | core | within-population cross-pollination | dry | 1 |
| 115 | Cor_3 | core | within-population cross-pollination | dry | 1 |
| 116 | Cor_3 | core | within-population cross-pollination | dry | 1 |
| 117 | Cor_3 | core | within-population cross-pollination | dry | 1 |
| 118 | Cor_3 | core | within-population cross-pollination | dry | 1 |
| 119 | Cor_3 | core | within-population cross-pollination | dry | 1 |
| 120 | Cor_3 | core | within-population cross-pollination | dry | 0 |
| 121 | Cor_3 | core | within-population cross-pollination | dry | 1 |
| 122 | Cor_3 | core | within-population cross-pollination | wet | 1 |
| 123 | Cor_3 | core | within-population cross-pollination | wet | 1 |
| 124 | Cor_3 | core | within-population cross-pollination | wet | 1 |
| 125 | Cor_3 | core | within-population cross-pollination | wet | 1 |
| 126 | Cor_3 | core | within-population cross-pollination | wet | 1 |
| 127 | Cor_3 | core | within-population cross-pollination | wet | 1 |
| 128 | Cor_3 | core | within-population cross-pollination | wet | 1 |
| 129 | Cor_3 | core | within-population cross-pollination | wet | 0 |
| 130 | Cor_3 | core | self-pollination | dry | 1 |
| 131 | Cor_3 | core | self-pollination | dry | 1 |
| 132 | Cor_3 | core | self-pollination | dry | 0 |
| 133 | Cor_3 | core | self-pollination | dry | 0 |
| 134 | Cor_3 | core | self-pollination | dry | 1 |
| 135 | Cor_3 | core | self-pollination | dry | 1 |
| 136 | Cor_3 | core | self-pollination | dry | 0 |
| 137 | Cor_3 | core | self-pollination | dry | 0 |
| 138 | Cor_3 | core | self-pollination | wet | 0 |
| 139 | Cor_3 | core | self-pollination | wet | 1 |
| 140 | Cor_3 | core | self-pollination | wet | 1 |
| 141 | Cor_3 | core | self-pollination | wet | 0 |
| 142 | Cor_3 | core | self-pollination | wet | 1 |
| 143 | Cor_3 | core | self-pollination | wet | 1 |
| 144 | Cor_3 | core | self-pollination | wet | 0 |
| 145 | Cor_3 | core | self-pollination | wet | 0 |
| 146 | Cor_4 | core | between-population cross-pollination | dry | 1 |
| 147 | Cor_4 | core | between-population cross-pollination | dry | 0 |
| 148 | Cor_4 | core | between-population cross-pollination | dry | 1 |
| 149 | Cor_4 | core | between-population cross-pollination | dry | 1 |
| 150 | Cor_4 | core | between-population cross-pollination | dry | 0 |
| 151 | Cor_4 | core | between-population cross-pollination | dry | 1 |
| 152 | Cor_4 | core | between-population cross-pollination | dry | 0 |
| 153 | Cor_4 | core | between-population cross-pollination | dry | 1 |
| 154 | Cor_4 | core | between-population cross-pollination | wet | 1 |
| 155 | Cor_4 | core | between-population cross-pollination | wet | 1 |
| 156 | Cor_4 | core | between-population cross-pollination | wet | 0 |
| 157 | Cor_4 | core | between-population cross-pollination | wet | 0 |
| 158 | Cor_4 | core | between-population cross-pollination | wet | 1 |
| 159 | Cor_4 | core | between-population cross-pollination | wet | 1 |
| 160 | Cor_4 | core | between-population cross-pollination | wet | 1 |
| 161 | Cor_4 | core | between-population cross-pollination | wet | 1 |
| 162 | Cor_4 | core | within-population cross-pollination | dry | 1 |
| 163 | Cor_4 | core | within-population cross-pollination | dry | 0 |
| 164 | Cor_4 | core | within-population cross-pollination | dry | 1 |
| 165 | Cor_4 | core | within-population cross-pollination | dry | 1 |
| 166 | Cor_4 | core | within-population cross-pollination | dry | 0 |
| 167 | Cor_4 | core | within-population cross-pollination | dry | 1 |
| 168 | Cor_4 | core | within-population cross-pollination | dry | 0 |
| 169 | Cor_4 | core | within-population cross-pollination | dry | 1 |
| 170 | Cor_4 | core | within-population cross-pollination | wet | 1 |
| 171 | Cor_4 | core | within-population cross-pollination | wet | 1 |
| 172 | Cor_4 | core | within-population cross-pollination | wet | 1 |
| 173 | Cor_4 | core | within-population cross-pollination | wet | 0 |
| 174 | Cor_4 | core | within-population cross-pollination | wet | 1 |
| 175 | Cor_4 | core | within-population cross-pollination | wet | 1 |
| 176 | Cor_4 | core | within-population cross-pollination | wet | 1 |
| 177 | Cor_4 | core | within-population cross-pollination | wet | 1 |
| 178 | Cor_4 | core | self-pollination | dry | 1 |
| 179 | Cor_4 | core | self-pollination | dry | 1 |
| 180 | Cor_4 | core | self-pollination | dry | 1 |
| 181 | Cor_4 | core | self-pollination | dry | 0 |
| 182 | Cor_4 | core | self-pollination | dry | 0 |
| 183 | Cor_4 | core | self-pollination | dry | 1 |
| 184 | Cor_4 | core | self-pollination | dry | 0 |
| 185 | Cor_4 | core | self-pollination | dry | 0 |
| 186 | Cor_4 | core | self-pollination | wet | 0 |
| 187 | Cor_4 | core | self-pollination | wet | 0 |
| 188 | Cor_4 | core | self-pollination | wet | 1 |
| 189 | Cor_4 | core | self-pollination | wet | 0 |
| 190 | Cor_4 | core | self-pollination | wet | 1 |
| 191 | Cor_4 | core | self-pollination | wet | 1 |
| 192 | Cor_4 | core | self-pollination | wet | 1 |
| 193 | Cor_4 | core | self-pollination | wet | 0 |
| 194 | Cor_5 | core | between-population cross-pollination | dry | 1 |
| 195 | Cor_5 | core | between-population cross-pollination | dry | 1 |
| 196 | Cor_5 | core | between-population cross-pollination | dry | 1 |
| 197 | Cor_5 | core | between-population cross-pollination | dry | 0 |
| 198 | Cor_5 | core | between-population cross-pollination | dry | 1 |
| 199 | Cor_5 | core | between-population cross-pollination | dry | 1 |
| 200 | Cor_5 | core | between-population cross-pollination | dry | 1 |
| 201 | Cor_5 | core | between-population cross-pollination | dry | 1 |
| 202 | Cor_5 | core | between-population cross-pollination | wet | 1 |
| 203 | Cor_5 | core | between-population cross-pollination | wet | 1 |
| 204 | Cor_5 | core | between-population cross-pollination | wet | 0 |
| 205 | Cor_5 | core | between-population cross-pollination | wet | 1 |
| 206 | Cor_5 | core | between-population cross-pollination | wet | 1 |
| 207 | Cor_5 | core | between-population cross-pollination | wet | 1 |
| 208 | Cor_5 | core | between-population cross-pollination | wet | 1 |
| 209 | Cor_5 | core | within-population cross-pollination | dry | 1 |
| 210 | Cor_5 | core | within-population cross-pollination | dry | 0 |
| 211 | Cor_5 | core | within-population cross-pollination | dry | 1 |
| 212 | Cor_5 | core | within-population cross-pollination | dry | 0 |
| 213 | Cor_5 | core | within-population cross-pollination | dry | 0 |
| 214 | Cor_5 | core | within-population cross-pollination | dry | 1 |
| 215 | Cor_5 | core | within-population cross-pollination | dry | 1 |
| 216 | Cor_5 | core | within-population cross-pollination | dry | 1 |
| 217 | Cor_5 | core | within-population cross-pollination | wet | 0 |
| 218 | Cor_5 | core | within-population cross-pollination | wet | 1 |
| 219 | Cor_5 | core | within-population cross-pollination | wet | 0 |
| 220 | Cor_5 | core | within-population cross-pollination | wet | 0 |
| 221 | Cor_5 | core | within-population cross-pollination | wet | 1 |
| 222 | Cor_5 | core | within-population cross-pollination | wet | 1 |
| 223 | Cor_5 | core | within-population cross-pollination | wet | 1 |
| 224 | Cor_5 | core | within-population cross-pollination | wet | 0 |
| 225 | Cor_5 | core | self-pollination | dry | 1 |
| 226 | Cor_5 | core | self-pollination | dry | 1 |
| 227 | Cor_5 | core | self-pollination | dry | 0 |
| 228 | Cor_5 | core | self-pollination | dry | 1 |
| 229 | Cor_5 | core | self-pollination | dry | 0 |
| 230 | Cor_5 | core | self-pollination | dry | 1 |
| 231 | Cor_5 | core | self-pollination | dry | 1 |
| 232 | Cor_5 | core | self-pollination | dry | 0 |
| 233 | Cor_5 | core | self-pollination | wet | 1 |
| 234 | Cor_5 | core | self-pollination | wet | 0 |
| 235 | Cor_5 | core | self-pollination | wet | 0 |
| 236 | Cor_5 | core | self-pollination | wet | 1 |
| 237 | Cor_5 | core | self-pollination | wet | 1 |
| 238 | Cor_5 | core | self-pollination | wet | 1 |
| 239 | Cor_5 | core | self-pollination | wet | 1 |
| 240 | Cor_5 | core | self-pollination | wet | 0 |
| 241 | Per_1 | Peripheral | between-population cross-pollination | dry | 1 |
| 242 | Per_1 | Peripheral | between-population cross-pollination | dry | 0 |
| 243 | Per_1 | Peripheral | between-population cross-pollination | dry | 0 |
| 244 | Per_1 | Peripheral | between-population cross-pollination | dry | 1 |
| 245 | Per_1 | Peripheral | between-population cross-pollination | dry | 1 |
| 246 | Per_1 | Peripheral | between-population cross-pollination | dry | 1 |
| 247 | Per_1 | Peripheral | between-population cross-pollination | dry | 1 |
| 248 | Per_1 | Peripheral | between-population cross-pollination | dry | 0 |
| 249 | Per_1 | Peripheral | between-population cross-pollination | wet | 1 |
| 250 | Per_1 | Peripheral | between-population cross-pollination | wet | 1 |
| 251 | Per_1 | Peripheral | between-population cross-pollination | wet | 1 |
| 252 | Per_1 | Peripheral | between-population cross-pollination | wet | 1 |
| 253 | Per_1 | Peripheral | between-population cross-pollination | wet | 0 |
| 254 | Per_1 | Peripheral | between-population cross-pollination | wet | 1 |
| 255 | Per_1 | Peripheral | between-population cross-pollination | wet | 1 |
| 256 | Per_1 | Peripheral | between-population cross-pollination | wet | 1 |
| 257 | Per_1 | Peripheral | within-population cross-pollination | dry | 0 |
| 258 | Per_1 | Peripheral | within-population cross-pollination | dry | 1 |
| 259 | Per_1 | Peripheral | within-population cross-pollination | dry | 0 |
| 260 | Per_1 | Peripheral | within-population cross-pollination | dry | 0 |
| 261 | Per_1 | Peripheral | within-population cross-pollination | dry | 1 |
| 262 | Per_1 | Peripheral | within-population cross-pollination | dry | 1 |
| 263 | Per_1 | Peripheral | within-population cross-pollination | dry | 0 |
| 264 | Per_1 | Peripheral | within-population cross-pollination | dry | 1 |
| 265 | Per_1 | Peripheral | within-population cross-pollination | wet | 1 |
| 266 | Per_1 | Peripheral | within-population cross-pollination | wet | 1 |
| 267 | Per_1 | Peripheral | within-population cross-pollination | wet | 0 |
| 268 | Per_1 | Peripheral | within-population cross-pollination | wet | 1 |
| 269 | Per_1 | Peripheral | within-population cross-pollination | wet | 1 |
| 270 | Per_1 | Peripheral | within-population cross-pollination | wet | 1 |
| 271 | Per_1 | Peripheral | within-population cross-pollination | wet | 1 |
| 272 | Per_1 | Peripheral | within-population cross-pollination | wet | 1 |
| 273 | Per_1 | Peripheral | self-pollination | dry | 0 |
| 274 | Per_1 | Peripheral | self-pollination | dry | 0 |
| 275 | Per_1 | Peripheral | self-pollination | dry | 1 |
| 276 | Per_1 | Peripheral | self-pollination | dry | 1 |
| 277 | Per_1 | Peripheral | self-pollination | dry | 0 |
| 278 | Per_1 | Peripheral | self-pollination | dry | 0 |
| 279 | Per_1 | Peripheral | self-pollination | dry | 1 |
| 280 | Per_1 | Peripheral | self-pollination | dry | 1 |
| 281 | Per_1 | Peripheral | self-pollination | wet | 1 |
| 282 | Per_1 | Peripheral | self-pollination | wet | 0 |
| 283 | Per_1 | Peripheral | self-pollination | wet | 1 |
| 284 | Per_1 | Peripheral | self-pollination | wet | 0 |
| 285 | Per_1 | Peripheral | self-pollination | wet | 0 |
| 286 | Per_1 | Peripheral | self-pollination | wet | 1 |
| 287 | Per_1 | Peripheral | self-pollination | wet | 1 |
| 288 | Per_1 | Peripheral | self-pollination | wet | 0 |
| 289 | Per_2 | Peripheral | between-population cross-pollination | dry | 1 |
| 290 | Per_2 | Peripheral | between-population cross-pollination | dry | 1 |
| 291 | Per_2 | Peripheral | between-population cross-pollination | dry | 1 |
| 292 | Per_2 | Peripheral | between-population cross-pollination | dry | 0 |
| 293 | Per_2 | Peripheral | between-population cross-pollination | dry | 0 |
| 294 | Per_2 | Peripheral | between-population cross-pollination | dry | 1 |
| 295 | Per_2 | Peripheral | between-population cross-pollination | dry | 1 |
| 296 | Per_2 | Peripheral | between-population cross-pollination | dry | 0 |
| 297 | Per_2 | Peripheral | between-population cross-pollination | wet | 0 |
| 298 | Per_2 | Peripheral | between-population cross-pollination | wet | 1 |
| 299 | Per_2 | Peripheral | between-population cross-pollination | wet | 0 |
| 300 | Per_2 | Peripheral | between-population cross-pollination | wet | 0 |
| 301 | Per_2 | Peripheral | between-population cross-pollination | wet | 1 |
| 302 | Per_2 | Peripheral | between-population cross-pollination | wet | 1 |
| 303 | Per_2 | Peripheral | between-population cross-pollination | wet | 1 |
| 304 | Per_2 | Peripheral | between-population cross-pollination | wet | 1 |
| 305 | Per_2 | Peripheral | within-population cross-pollination | dry | 1 |
| 306 | Per_2 | Peripheral | within-population cross-pollination | dry | 1 |
| 307 | Per_2 | Peripheral | within-population cross-pollination | dry | 0 |
| 308 | Per_2 | Peripheral | within-population cross-pollination | dry | 1 |
| 309 | Per_2 | Peripheral | within-population cross-pollination | dry | 0 |
| 310 | Per_2 | Peripheral | within-population cross-pollination | dry | 1 |
| 311 | Per_2 | Peripheral | within-population cross-pollination | dry | 1 |
| 312 | Per_2 | Peripheral | within-population cross-pollination | dry | 1 |
| 313 | Per_2 | Peripheral | within-population cross-pollination | wet | 1 |
| 314 | Per_2 | Peripheral | within-population cross-pollination | wet | 0 |
| 315 | Per_2 | Peripheral | within-population cross-pollination | wet | 1 |
| 316 | Per_2 | Peripheral | within-population cross-pollination | wet | 0 |
| 317 | Per_2 | Peripheral | within-population cross-pollination | wet | 1 |
| 318 | Per_2 | Peripheral | within-population cross-pollination | wet | 1 |
| 319 | Per_2 | Peripheral | within-population cross-pollination | wet | 1 |
| 320 | Per_2 | Peripheral | within-population cross-pollination | wet | 0 |
| 321 | Per_2 | Peripheral | self-pollination | dry | 1 |
| 322 | Per_2 | Peripheral | self-pollination | dry | 0 |
| 323 | Per_2 | Peripheral | self-pollination | dry | 0 |
| 324 | Per_2 | Peripheral | self-pollination | dry | 0 |
| 325 | Per_2 | Peripheral | self-pollination | dry | 1 |
| 326 | Per_2 | Peripheral | self-pollination | dry | 1 |
| 327 | Per_2 | Peripheral | self-pollination | dry | 1 |
| 328 | Per_2 | Peripheral | self-pollination | dry | 0 |
| 329 | Per_2 | Peripheral | self-pollination | wet | 1 |
| 330 | Per_2 | Peripheral | self-pollination | wet | 0 |
| 331 | Per_2 | Peripheral | self-pollination | wet | 0 |
| 332 | Per_2 | Peripheral | self-pollination | wet | 1 |
| 333 | Per_2 | Peripheral | self-pollination | wet | 1 |
| 334 | Per_2 | Peripheral | self-pollination | wet | 1 |
| 335 | Per_2 | Peripheral | self-pollination | wet | 1 |
| 336 | Per_2 | Peripheral | self-pollination | wet | 0 |
| 337 | Per_3 | Peripheral | between-population cross-pollination | dry | 1 |
| 338 | Per_3 | Peripheral | between-population cross-pollination | dry | 1 |
| 339 | Per_3 | Peripheral | between-population cross-pollination | dry | 0 |
| 340 | Per_3 | Peripheral | between-population cross-pollination | dry | 0 |
| 341 | Per_3 | Peripheral | between-population cross-pollination | dry | 0 |
| 342 | Per_3 | Peripheral | between-population cross-pollination | dry | 0 |
| 343 | Per_3 | Peripheral | between-population cross-pollination | dry | 1 |
| 344 | Per_3 | Peripheral | between-population cross-pollination | dry | 0 |
| 345 | Per_3 | Peripheral | between-population cross-pollination | wet | 1 |
| 346 | Per_3 | Peripheral | between-population cross-pollination | wet | 0 |
| 347 | Per_3 | Peripheral | between-population cross-pollination | wet | 0 |
| 348 | Per_3 | Peripheral | between-population cross-pollination | wet | 1 |
| 349 | Per_3 | Peripheral | between-population cross-pollination | wet | 1 |
| 350 | Per_3 | Peripheral | between-population cross-pollination | wet | 1 |
| 351 | Per_3 | Peripheral | between-population cross-pollination | wet | 1 |
| 352 | Per_3 | Peripheral | between-population cross-pollination | wet | 1 |
| 353 | Per_3 | Peripheral | within-population cross-pollination | dry | 0 |
| 354 | Per_3 | Peripheral | within-population cross-pollination | dry | 0 |
| 355 | Per_3 | Peripheral | within-population cross-pollination | dry | 0 |
| 356 | Per_3 | Peripheral | within-population cross-pollination | dry | 1 |
| 357 | Per_3 | Peripheral | within-population cross-pollination | dry | 1 |
| 358 | Per_3 | Peripheral | within-population cross-pollination | dry | 1 |
| 359 | Per_3 | Peripheral | within-population cross-pollination | dry | 1 |
| 360 | Per_3 | Peripheral | within-population cross-pollination | dry | 1 |
| 361 | Per_3 | Peripheral | within-population cross-pollination | wet | 1 |
| 362 | Per_3 | Peripheral | within-population cross-pollination | wet | 0 |
| 363 | Per_3 | Peripheral | within-population cross-pollination | wet | 1 |
| 364 | Per_3 | Peripheral | within-population cross-pollination | wet | 1 |
| 365 | Per_3 | Peripheral | within-population cross-pollination | wet | 1 |
| 366 | Per_3 | Peripheral | within-population cross-pollination | wet | 0 |
| 367 | Per_3 | Peripheral | within-population cross-pollination | wet | 1 |
| 368 | Per_3 | Peripheral | within-population cross-pollination | wet | 1 |
| 369 | Per_3 | Peripheral | self-pollination | dry | 1 |
| 370 | Per_3 | Peripheral | self-pollination | dry | 1 |
| 371 | Per_3 | Peripheral | self-pollination | dry | 0 |
| 372 | Per_3 | Peripheral | self-pollination | dry | 0 |
| 373 | Per_3 | Peripheral | self-pollination | dry | 0 |
| 374 | Per_3 | Peripheral | self-pollination | dry | 1 |
| 375 | Per_3 | Peripheral | self-pollination | dry | 0 |
| 376 | Per_3 | Peripheral | self-pollination | dry | 0 |
| 377 | Per_3 | Peripheral | self-pollination | wet | 0 |
| 378 | Per_3 | Peripheral | self-pollination | wet | 0 |
| 379 | Per_3 | Peripheral | self-pollination | wet | 0 |
| 380 | Per_3 | Peripheral | self-pollination | wet | 1 |
| 381 | Per_3 | Peripheral | self-pollination | wet | 1 |
| 382 | Per_3 | Peripheral | self-pollination | wet | 1 |
| 383 | Per_3 | Peripheral | self-pollination | wet | 1 |
| 384 | Per_3 | Peripheral | self-pollination | wet | 0 |
| 385 | Per_4 | Peripheral | between-population cross-pollination | dry | 1 |
| 386 | Per_4 | Peripheral | between-population cross-pollination | dry | 0 |
| 387 | Per_4 | Peripheral | between-population cross-pollination | dry | 0 |
| 388 | Per_4 | Peripheral | between-population cross-pollination | dry | 0 |
| 389 | Per_4 | Peripheral | between-population cross-pollination | dry | 0 |
| 390 | Per_4 | Peripheral | between-population cross-pollination | dry | 0 |
| 391 | Per_4 | Peripheral | between-population cross-pollination | dry | 1 |
| 392 | Per_4 | Peripheral | between-population cross-pollination | dry | 0 |
| 393 | Per_4 | Peripheral | between-population cross-pollination | wet | 1 |
| 394 | Per_4 | Peripheral | between-population cross-pollination | wet | 1 |
| 395 | Per_4 | Peripheral | between-population cross-pollination | wet | 1 |
| 396 | Per_4 | Peripheral | between-population cross-pollination | wet | 1 |
| 397 | Per_4 | Peripheral | between-population cross-pollination | wet | 1 |
| 398 | Per_4 | Peripheral | between-population cross-pollination | wet | 1 |
| 399 | Per_4 | Peripheral | between-population cross-pollination | wet | 1 |
| 400 | Per_4 | Peripheral | between-population cross-pollination | wet | 1 |
| 401 | Per_4 | Peripheral | within-population cross-pollination | dry | 1 |
| 402 | Per_4 | Peripheral | within-population cross-pollination | dry | 1 |
| 403 | Per_4 | Peripheral | within-population cross-pollination | dry | 0 |
| 404 | Per_4 | Peripheral | within-population cross-pollination | dry | 1 |
| 405 | Per_4 | Peripheral | within-population cross-pollination | dry | 0 |
| 406 | Per_4 | Peripheral | within-population cross-pollination | dry | 1 |
| 407 | Per_4 | Peripheral | within-population cross-pollination | dry | 1 |
| 408 | Per_4 | Peripheral | within-population cross-pollination | dry | 0 |
| 409 | Per_4 | Peripheral | within-population cross-pollination | wet | 0 |
| 410 | Per_4 | Peripheral | within-population cross-pollination | wet | 0 |
| 411 | Per_4 | Peripheral | within-population cross-pollination | wet | 1 |
| 412 | Per_4 | Peripheral | within-population cross-pollination | wet | 1 |
| 413 | Per_4 | Peripheral | within-population cross-pollination | wet | 1 |
| 414 | Per_4 | Peripheral | within-population cross-pollination | wet | 1 |
| 415 | Per_4 | Peripheral | within-population cross-pollination | wet | 1 |
| 416 | Per_4 | Peripheral | within-population cross-pollination | wet | 1 |
| 417 | Per_4 | Peripheral | self-pollination | dry | 0 |
| 418 | Per_4 | Peripheral | self-pollination | dry | 1 |
| 419 | Per_4 | Peripheral | self-pollination | dry | 0 |
| 420 | Per_4 | Peripheral | self-pollination | dry | 0 |
| 421 | Per_4 | Peripheral | self-pollination | dry | 0 |
| 422 | Per_4 | Peripheral | self-pollination | dry | 1 |
| 423 | Per_4 | Peripheral | self-pollination | dry | 1 |
| 424 | Per_4 | Peripheral | self-pollination | dry | 1 |
| 425 | Per_4 | Peripheral | self-pollination | wet | 0 |
| 426 | Per_4 | Peripheral | self-pollination | wet | 1 |
| 427 | Per_4 | Peripheral | self-pollination | wet | 0 |
| 428 | Per_4 | Peripheral | self-pollination | wet | 1 |
| 429 | Per_4 | Peripheral | self-pollination | wet | 0 |
| 430 | Per_4 | Peripheral | self-pollination | wet | 1 |
| 431 | Per_4 | Peripheral | self-pollination | wet | 1 |
| 432 | Per_4 | Peripheral | self-pollination | wet | 0 |
| 433 | Per_5 | Peripheral | between-population cross-pollination | dry | 0 |
| 434 | Per_5 | Peripheral | between-population cross-pollination | dry | 0 |
| 435 | Per_5 | Peripheral | between-population cross-pollination | dry | 1 |
| 436 | Per_5 | Peripheral | between-population cross-pollination | dry | 0 |
| 437 | Per_5 | Peripheral | between-population cross-pollination | dry | 1 |
| 438 | Per_5 | Peripheral | between-population cross-pollination | dry | 0 |
| 439 | Per_5 | Peripheral | between-population cross-pollination | dry | 0 |
| 440 | Per_5 | Peripheral | between-population cross-pollination | dry | 1 |
| 441 | Per_5 | Peripheral | between-population cross-pollination | wet | 0 |
| 442 | Per_5 | Peripheral | between-population cross-pollination | wet | 1 |
| 443 | Per_5 | Peripheral | between-population cross-pollination | wet | 1 |
| 444 | Per_5 | Peripheral | between-population cross-pollination | wet | 1 |
| 445 | Per_5 | Peripheral | between-population cross-pollination | wet | 1 |
| 446 | Per_5 | Peripheral | between-population cross-pollination | wet | 1 |
| 447 | Per_5 | Peripheral | between-population cross-pollination | wet | 1 |
| 448 | Per_5 | Peripheral | between-population cross-pollination | wet | 1 |
| 449 | Per_5 | Peripheral | within-population cross-pollination | dry | 0 |
| 450 | Per_5 | Peripheral | within-population cross-pollination | dry | 1 |
| 451 | Per_5 | Peripheral | within-population cross-pollination | dry | 0 |
| 452 | Per_5 | Peripheral | within-population cross-pollination | dry | 1 |
| 453 | Per_5 | Peripheral | within-population cross-pollination | dry | 1 |
| 454 | Per_5 | Peripheral | within-population cross-pollination | dry | 1 |
| 455 | Per_5 | Peripheral | within-population cross-pollination | dry | 0 |
| 456 | Per_5 | Peripheral | within-population cross-pollination | dry | 1 |
| 457 | Per_5 | Peripheral | within-population cross-pollination | wet | 0 |
| 458 | Per_5 | Peripheral | within-population cross-pollination | wet | 1 |
| 459 | Per_5 | Peripheral | within-population cross-pollination | wet | 0 |
| 460 | Per_5 | Peripheral | within-population cross-pollination | wet | 0 |
| 461 | Per_5 | Peripheral | within-population cross-pollination | wet | 1 |
| 462 | Per_5 | Peripheral | within-population cross-pollination | wet | 0 |
| 463 | Per_5 | Peripheral | within-population cross-pollination | wet | 1 |
| 464 | Per_5 | Peripheral | within-population cross-pollination | wet | 1 |
| 465 | Per_5 | Peripheral | self-pollination | dry | 1 |
| 466 | Per_5 | Peripheral | self-pollination | dry | 0 |
| 467 | Per_5 | Peripheral | self-pollination | dry | 1 |
| 468 | Per_5 | Peripheral | self-pollination | dry | 0 |
| 469 | Per_5 | Peripheral | self-pollination | dry | 0 |
| 470 | Per_5 | Peripheral | self-pollination | dry | 1 |
| 471 | Per_5 | Peripheral | self-pollination | dry | 1 |
| 472 | Per_5 | Peripheral | self-pollination | dry | 0 |
| 473 | Per_5 | Peripheral | self-pollination | wet | 0 |
| 474 | Per_5 | Peripheral | self-pollination | wet | 1 |
| 475 | Per_5 | Peripheral | self-pollination | wet | 1 |
| 476 | Per_5 | Peripheral | self-pollination | wet | 1 |
| 477 | Per_5 | Peripheral | self-pollination | wet | 1 |
| 478 | Per_5 | Peripheral | self-pollination | wet | 1 |
| 479 | Per_5 | Peripheral | self-pollination | wet | 1 |
| 480 | Per_5 | Peripheral | self-pollination | wet | 1 |
