# Supplementary material for: Do marginal plant populations enhance the fitness of larger core units under ongoing climate change? Empirical insights from a rare carnation
Source: AoB Plants. 2022 May 12;14(3):plac022. doi: 10.1093/aobpla/plac022 (PMC9167561; doi:10.1093/aobpla/plac022)

Appendix S4. Figures showing average germination, growth, and survival rates expressed by each maternal family under different pollination and stress treatments.

Figure S4.1. Mean germination rate recorded in each family under different pollination and stress protocols.

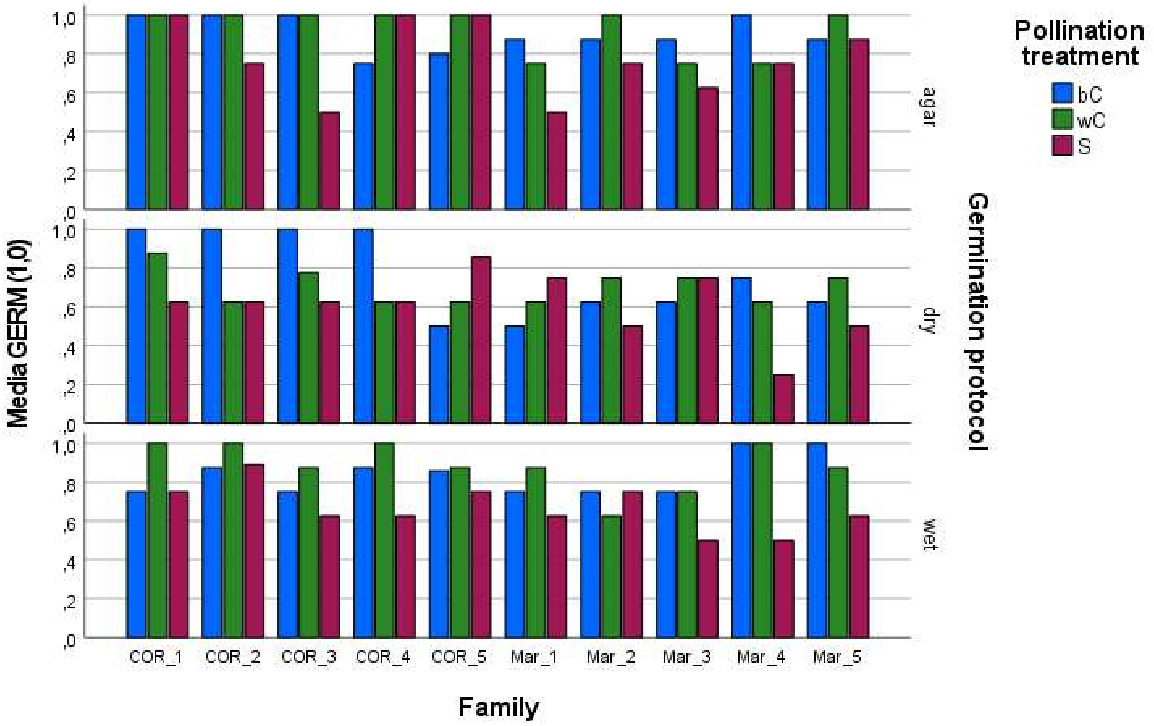

Figure S4.2 Mean radicle length recorded in each family under different pollination and stress treatments.

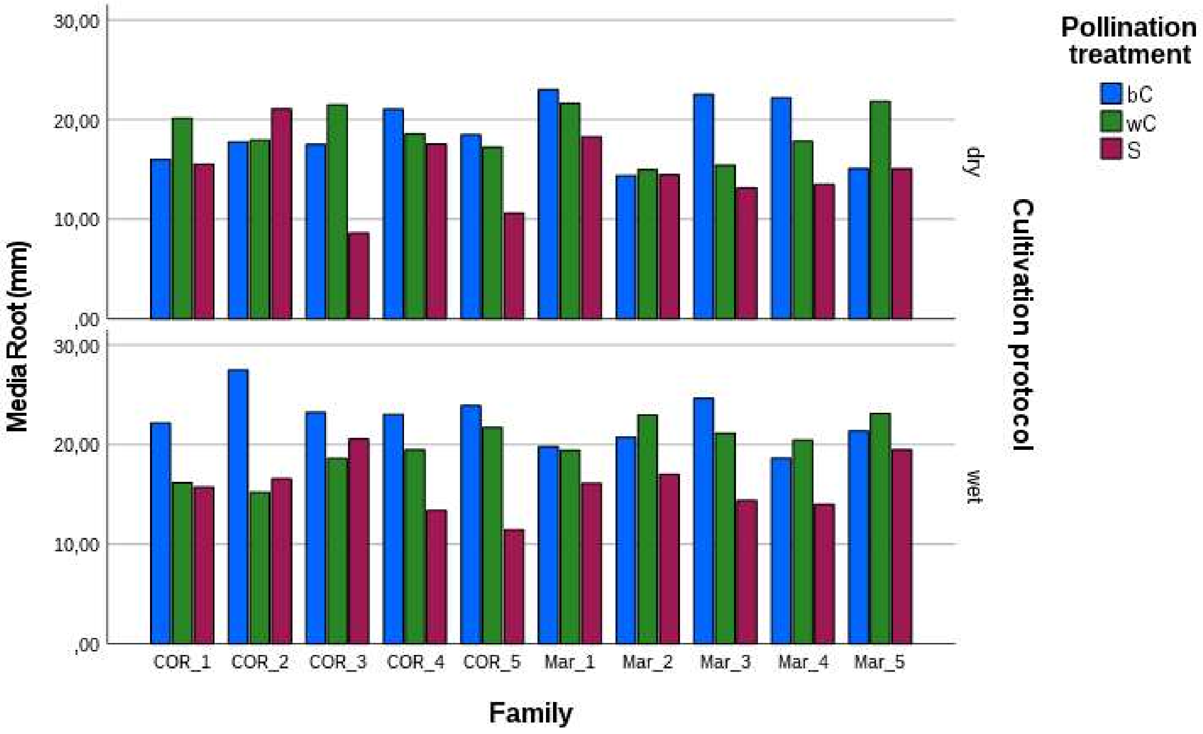

Figure S4.3 Mean shoot size recorded in each family under different pollination and stress treatments.

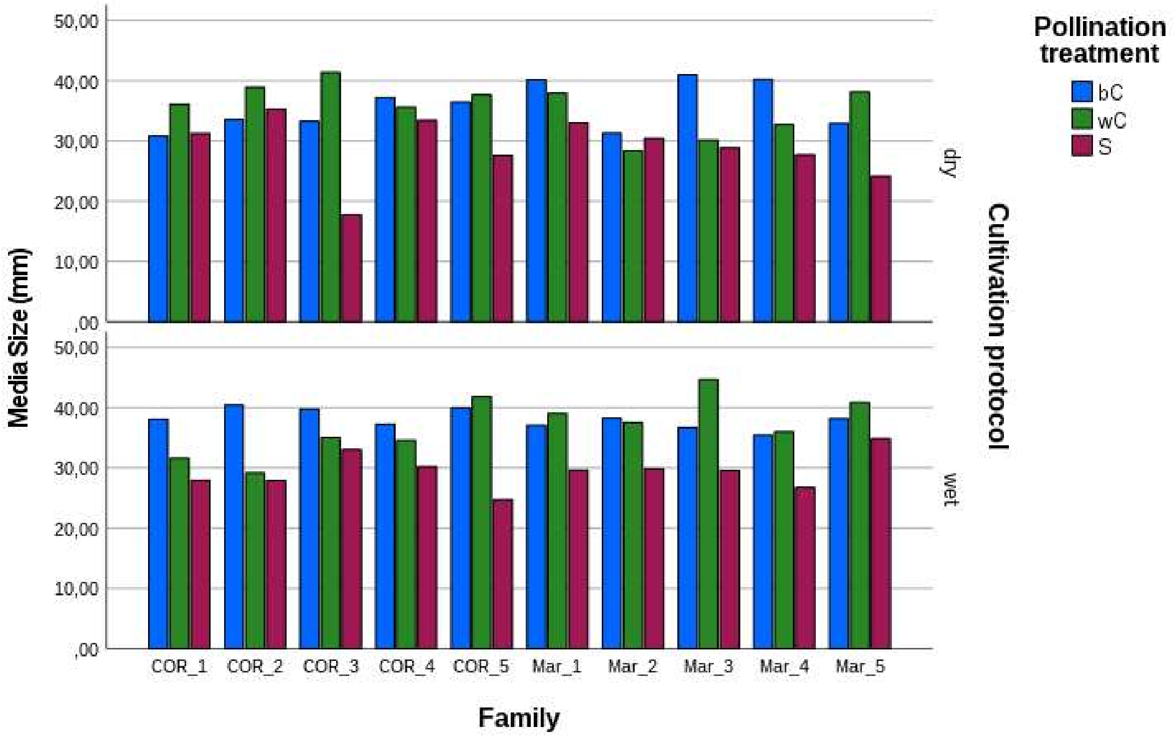

Figure S4.4 Mean survival rate recorded in each family under different pollination and stress treatments.

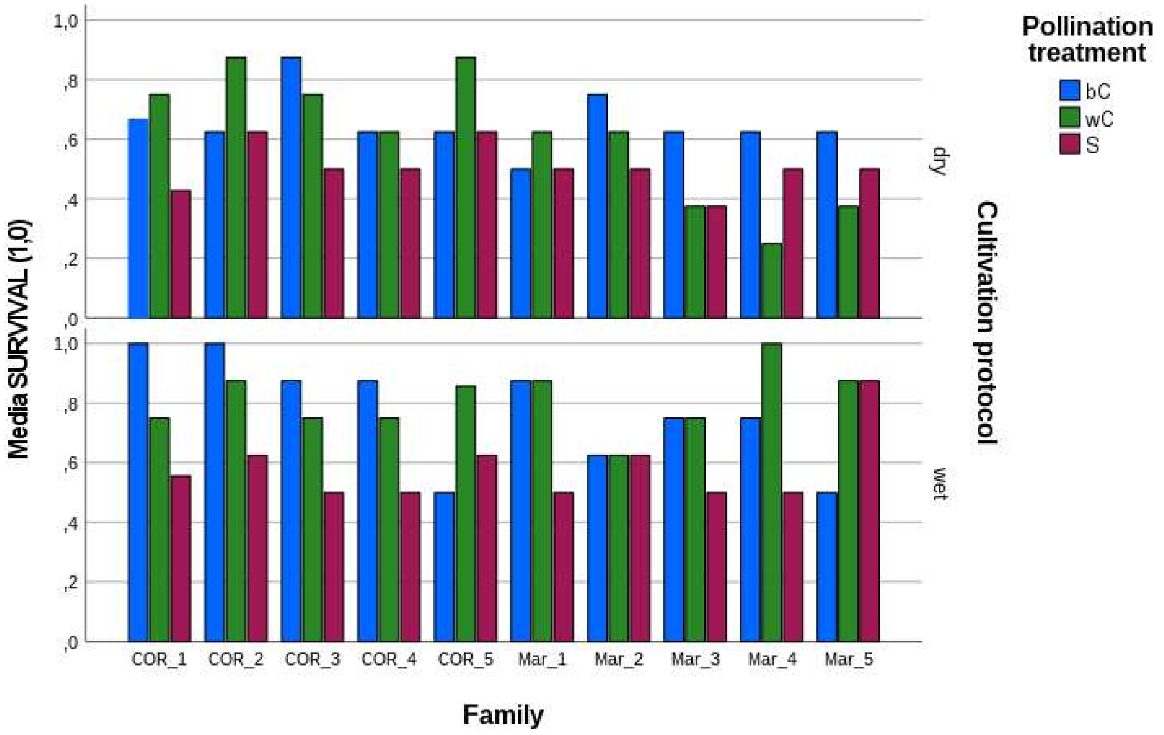

Supplement: plac022_suppl_Supplementary_Appendix_S4 [file plac022_suppl_supplementary_appendix_s4.pdf]
